# A functionally impaired missense variant identified in French Canadian families implicates *FANCI* as a candidate ovarian cancer-predisposing gene

**DOI:** 10.1186/s13073-021-00998-5

**Published:** 2021-12-03

**Authors:** Caitlin T. Fierheller, Laure Guitton-Sert, Wejdan M. Alenezi, Timothée Revil, Kathleen K. Oros, Yuandi Gao, Karine Bedard, Suzanna L. Arcand, Corinne Serruya, Supriya Behl, Liliane Meunier, Hubert Fleury, Eleanor Fewings, Deepak N. Subramanian, Javad Nadaf, Jeffrey P. Bruce, Rachel Bell, Diane Provencher, William D. Foulkes, Zaki El Haffaf, Anne-Marie Mes-Masson, Jacek Majewski, Trevor J. Pugh, Marc Tischkowitz, Paul A. James, Ian G. Campbell, Celia M. T. Greenwood, Jiannis Ragoussis, Jean-Yves Masson, Patricia N. Tonin

**Affiliations:** 1 Department of Human Genetics, McGill University, Montreal, Quebec Canada; 2 Cancer Research Program, Centre for Translational Biology, The Research Institute of the McGill University Health Centre, 1001 Decarie Boulevard, Montreal, Quebec H4A 3 J1 Canada; 3 Genome Stability Laboratory, CHU de Québec-Université Laval Research Center, Oncology Division, Quebec City, Quebec Canada; 4 Department of Molecular Biology, Medical Biochemistry and Pathology, Laval University Cancer Research Center, Quebec City, Quebec Canada; 5 Department of Medical Laboratory Technology, Taibah University, Medina, Saudi Arabia; 6 McGill Genome Centre, McGill University, Montreal, Quebec Canada; 7 Lady Davis Institute for Medical Research, Jewish General Hospital, Montreal, Quebec Canada; 8 Laboratoire de Diagnostic Moléculaire, Centre Hospitalier de l’Université de Montréal (CHUM), Montreal, Quebec Canada; 9 Département de pathologie et biologie cellulaire, Université de Montréal, Montreal, Quebec Canada; 10 Centre de recherche du Centre hospitalier de l’Université de Montréal and Institut du cancer de Montréal, Montreal, Quebec Canada; 11 Department of Medical Genetics, National Institute for Health Research Cambridge Biomedical Research Centre, University of Cambridge, Cambridge, UK; 12 Cancer Genetics Laboratory, Peter MacCallum Cancer Centre, Melbourne, Victoria Australia; 13 Princess Margaret Cancer Centre, University Health Network, Toronto, Ontario Canada; 14 Division of Gynecologic Oncology, Université de Montréal, Montreal, Quebec Canada; 15 Department of Medicine, McGill University, Montreal, Quebec Canada; 16 Centre de recherche du Centre Hospitalier de l’Université de Montréal, Montreal, Quebec Canada; 17 Department of Medicine, Université de Montréal, Montreal, Quebec Canada; 18 Department of Medical Biophysics, University of Toronto, Toronto, Ontario Canada; 19 Ontario Institute for Cancer Research, Toronto, Ontario Canada; 20 Sir Peter MacCallum Department of Oncology, University of Melbourne, Melbourne, Victoria Australia; 21 The Parkville Familial Cancer Centre, Peter MacCallum Cancer Centre and The Royal Melbourne Hospital, Melbourne, Victoria Australia; 22 Gerald Bronfman Department of Oncology, McGill University, Montreal, Quebec Canada; 23 Department of Epidemiology, Biostatistics & Occupational Health, McGill University, Montreal, Quebec Canada

**Keywords:** *FANCI*, Ovarian cancer, Cancer-predisposing gene, Whole exome sequencing, Tissue microarray, Protein expression, DNA repair, Fanconi anaemia pathway, Familial aggregation of cancer, Hereditary cancer

## Abstract

**Background:**

Familial ovarian cancer (OC) cases not harbouring pathogenic variants in either of the *BRCA1* and *BRCA2* OC-predisposing genes, which function in homologous recombination (HR) of DNA, could involve pathogenic variants in other DNA repair pathway genes.

**Methods:**

Whole exome sequencing was used to identify rare variants in HR genes in a *BRCA1* and *BRCA2* pathogenic variant negative OC family of French Canadian (FC) ancestry, a population exhibiting genetic drift. OC cases and cancer-free individuals from FC and non-FC populations were investigated for carrier frequency of *FANCI* c.1813C>T; p.L605F, the top-ranking candidate. Gene and protein expression were investigated in cancer cell lines and tissue microarrays, respectively.

**Results:**

In FC subjects, c.1813C>T was more common in familial (7.1%, 3/42) than sporadic (1.6%, 7/439) OC cases (*P* = 0.048). Carriers were detected in 2.5% (74/2950) of cancer-free females though female/male carriers were more likely to have a first-degree relative with OC (121/5249, 2.3%; Spearman correlation = 0.037; *P* = 0.011), suggesting a role in risk. Many of the cancer-free females had host factors known to reduce risk to OC which could influence cancer risk in this population. There was an increased carrier frequency of *FANCI* c.1813C>T in *BRCA1* and *BRCA2* pathogenic variant negative OC families, when including the discovery family, compared to cancer-free females (3/23, 13%; OR = 5.8; 95%CI = 1.7–19; *P* = 0.005). In non-FC subjects, 10 candidate *FANCI* variants were identified in 4.1% (21/516) of Australian OC cases negative for pathogenic variants in *BRCA1* and *BRCA2*, including 10 carriers of *FANCI* c.1813C>T. Candidate variants were significantly more common in familial OC than in sporadic OC (*P* = 0.04). Localization of FANCD2, part of the FANCI-FANCD2 (ID2) binding complex in the Fanconi anaemia (FA) pathway, to sites of induced DNA damage was severely impeded in cells expressing the p.L605F isoform. This isoform was expressed at a reduced level, destabilized by DNA damaging agent treatment in both HeLa and OC cell lines, and exhibited sensitivity to cisplatin but not to a poly (ADP-ribose) polymerase inhibitor. By tissue microarray analyses, FANCI protein was consistently expressed in fallopian tube epithelial cells and only expressed at low-to-moderate levels in 88% (83/94) of OC samples.

**Conclusions:**

This is the first study to describe candidate OC variants in *FANCI*, a member of the ID2 complex of the FA DNA repair pathway. Our data suggest that pathogenic *FANCI* variants may modify OC risk in cancer families.

**Supplementary Information:**

The online version contains supplementary material available at 10.1186/s13073-021-00998-5.

## Background

Ovarian cancer (OC), with an overall 5-year survival rate of 40%, is the leading cause of death in women with gynecologic cancer [1]. The overall lifetime risk for OC in the North American population is 1.3% [1]. However, twin studies suggest that 22% of OC risk can be attributed to heritable factors [2] and having an affected first-degree relative confers a 3–7-fold increase in risk to this disease [3, 4]. Carriers that are heterozygous for pathogenic variants in *BRCA1 (FANCS)* or *BRCA2 (FANCD1)* have an estimated lifetime risk for OC of 17–44% (by age 80 years), depending on the gene mutated [5]. Pathogenic *BRCA1* and *BRCA2* variants have been reported in 65–85% of cancer syndromes featuring high-grade serous ovarian carcinoma (HGSC) [6], the most common histopathological subtype of epithelial OC [7], and in 10–20% of HGSC cases regardless of age at diagnosis [8]. Identifying carriers of *BRCA1* and *BRCA2* pathogenic variants for cancer prevention (prophylactic surgery [9, 10]) and management of OC using new therapies (e.g. poly (ADP-ribose) polymerase inhibitors (PARPi) [11–16]) is being offered in medical genetic and gynecologic oncology settings.

New cancer-predisposing gene (CPG) candidates have been investigated with a focus on members of the Fanconi anaemia (FA) DNA repair pathway involving BRCA1 and BRCA2 function. The most promising new OC-predisposing genes are from reports of heterozygous carriers of candidate variants in *BRIP1 (FANCJ)* [17, 18], *RAD51C (FANCO)* [19–22], and *RAD51D* [23]. In cancer families, carriers of pathogenic *RAD51C* and *RAD51D* variants have been estimated to have cumulative risks to age 80 of 11% (95% confidence interval (CI) = 6–21) and 13% (95%CI = 7–23), respectively, for OC [24]. Collectively, carriers of pathogenic variants in these genes do not account for a large proportion of familial OC and breast cancer (BC) cases that have not been attributed to the known CPGs. Therefore, it is possible that new CPGs conferring risk to OC have yet to be discovered.

The low incidence of OC, rarity of pathogenic variants in each proposed CPG, and genetic heterogeneity of the general population pose major challenges in finding new OC-predisposing genes. An attractive strategy for finding additional CPGs focuses on the investigation of demographically (ethnically or geographically) defined populations that have a history of founder effects. Due to a relatively few number of ancestors, rapid expansion and geographic isolation during 1608~1760 of the small founding immigrant French population of Quebec from Europe (EUR), a loss of genetic variation has occurred resulting in subsequent waves of expansion of carriers of specific variants [25–29]. As French Canadians (FC) are more likely to harbour frequently occurring germline pathogenic variants, candidate variants for OC may be readily identified by sequencing familial cases and/or by comparing allele frequencies in cancer cases versus cancer-free controls in contrast to studies involving the general population due to allelic heterogeneity [25, 26]. Though 42 different pathogenic *BRCA1* or *BRCA2* variants have been identified in FC cancer families of Quebec, five recurrent pathogenic variants account for 84% of all mutation-positive BC and/or OC families [30]. This is in contrast to the over 2000 different pathogenic *BRCA1* and *BRCA2* variants reported for undefined populations [31]. Specific pathogenic variants in *PALB2* (*FANCN*—c.2323C>T; p.Q775X) [32] and *RAD51D* (c.620C>T; p.S207L) [33] have also been identified in FC BC and HGSC cases, respectively.

Using whole exome sequencing (WES), we identified carriers of the *FANCI* c.1813C>T; p.L605F missense variant in a *BRCA1* and *BRCA2* pathogenic variant negative FC OC family. We investigated this variant based on a candidate gene approach as *FANCI* is the FA Complementation Group I gene, which is an essential member of the FA-homologous recombination (HR) pathway that repairs interstrand crosslink (ICL) DNA damage and acts as the molecular switch to activate this pathway [34–38].

To evaluate the potential pathogenicity of *FANCI* c.1813C>T; p.L605F, we applied a strategy that took advantage of the observed genetic drift in the FC population by investigating its allele frequency in FC OC and cancer-free subjects. We performed in cellulo (HeLa and OC cell lines) and in vitro experiments to investigate the functional effects of the encoded p.L605F isoform and its response to therapies used in the treatment of OC. We also investigated FANCI expression in HGSC and normal tissues. Lastly, we investigated Canadian non-FC (CDN) and Australian (AUS) cancer cases for rare candidate *FANCI* variants.

## Methods

### Study subjects

Information about all study subjects obtained from various biobanking resources can be found in Additional file 1: Table S1.

The FC cancer samples were obtained from Réseau de recherche sur le cancer (RRCancer) Tumour and Data biobank. The OC samples from this biobank derived its collection from patients attending a major gynecologic oncology hospital centre in the province of Quebec. This centre largely services FCs, where it is estimated that 85% of samples come from participants who self-identify as FC [39]. Samples within this collection with a familial history of OC and/or BC have been extensively studied, where the majority self-report grandparental FC ancestry of index cancer affected cases [30, 40, 41]. The allele frequency of *FANCI* c.1813C>T was determined by investigating selected index OC or BC cases, defined based on family history of OC and/or BC or sporadic disease where cases were not selected based on family history of cancer, where all were self-reported FC ancestry as previously described [30, 40, 41] (see Additional file 1: Table S1). These cases were mostly ascertained over a 20-year period from the early 1990s to 2004. OC families had at least two epithelial OC cases within first-, second-, or third-degree relatives and the average age of diagnosis was 50 years (range 24–77). Hereditary breast and ovarian cancer (HBOC) families had at least two invasive BC cases diagnosed under the age of 65 and one epithelial OC case in first-, second-, or third-degree relatives and the average age of diagnosis was 43.7 years (range 18–65). Hereditary breast cancer (HBC) families had at least three invasive BC cases diagnosed under the age 65 in first-, second-, or third-degree relatives and the average age of diagnosis was 44.6 years (range 22–65). All first-, second-, and third-degree relations needed to be within the same branch of the family. The *FANCI* locus was investigated in available WES data from a subset of 157 OC or BC cases of FC ancestry (see Additional file 1: Table S1). Sporadic BC cases were diagnosed with invasive BC before the age of 70 (average = 52.7, range 25–69) [42]. We cannot exclude the possibility that some cases occurred in more than one study group: based on RRCancer biobanking sample number, OC cases from at least 13 families were also found in pedigrees from BC cases that were genotyped from the familial HBOC study group.

Carrier frequencies of candidate variants were investigated in cancer-free FC study subjects using genotyping data obtained from CARTaGENE [43], a resource containing biological samples, genetic and health data for up to 43,000 adult residents in Quebec. The subjects investigated were recruited between 2009 and 2014, and had an overall average age of 54.7 years (range 39–71) [43] and included 2950 females (average age = 54.3 years; range 39–71) and 2299 males (average age = 55.3 years; range 39–70). Selection criteria for individuals with genotyping data are biased towards individuals with higher quantity of health data (see Additional file 1: Table S2). Individuals were defined as FC if they were born in the province of Quebec, their parents and all four grandparents were born in Canada, and French was the first language learned.

Variants in the *FANCI* locus were investigated in available OC Association Consortium (OCAC) and BC Association Consortium (BCAC) data. These study groups and accompanying genotyping data have been described elsewhere [44–46]. Data from 25,509 epithelial OC cases (22,406 invasive cancer) and 40,491 controls of EUR ancestry [44] were available from OCAC, including those for histopathological subtypes for the entire cohort as have been previously reported [44]. Data from 46,785 BC cases and 42,892 controls of EUR ancestry [45, 46] were available from BCAC.

The *FANCI* locus was investigated in the AUS population from available germline sequencing data derived from WES analysis of HGSC cases as previously described [47]. Briefly, all AUS cases had ovarian, fallopian tube, or peritoneal cancer (*n* = 516) and did not carry pathogenic variants in *BRCA1* and/or *BRCA2* (see Additional file 1: Table S1). Genetic data from AUS controls (*n* = 4878) were available from the lifepool project as previously described [48].

The *FANCI* locus was investigated in germline sequencing data available from other non-FC CDN study groups comprised of female subjects with OC, BC, or pancreatic cancer (*n* = 63) who were recruited from health care research centres in the province of Quebec (Additional file 1: Table S1). All recruited individuals had a strong family history of BC. A BRCAPro score [49], which is based on studies of Ashkenazi Jewish and EUR ancestry individuals, was generated to predict the likelihood of families carrying pathogenic variants in *BRCA1* or *BRCA2*. Individuals with a BRCAPro score of > 10%, but with no pathogenic variants in these genes were selected. Of this set, 14 individuals were of Ashkenazi Jewish ancestry.

Kaplan-Meier curves for overall survival analysis was performed using available gene expression data from 35 cancer types (*n* = 12,373, including *n* = 425 OC cases), from The Cancer Genome Atlas (TCGA) Pan-Cancer data set of the TGCA Project and was obtained from (Additional file 1: Table S1) University of California Santa Cruz Xena Browser [50]. The *FANCI* locus was investigated in germline sequencing data available for 412 Pan-Cancer OC cases downloaded from TCGA. Characteristics for TCGA samples are available via the National Cancer Institute Genomic Data Commons and cBioPortal for Cancer Genomics.

To further protect the anonymity of study subjects, all samples were assigned a unique identifier and pedigrees were modified. This project has received approval from The McGill University Health Centre (MUHC) REB (MP-37-2019-4783 and 2017-2722). All participants provided informed consent and the research conformed to the principles of the Helsinki Declaration.

### Identification of candidate *FANCI* c.1813C>T variant

**Fig. 1:**
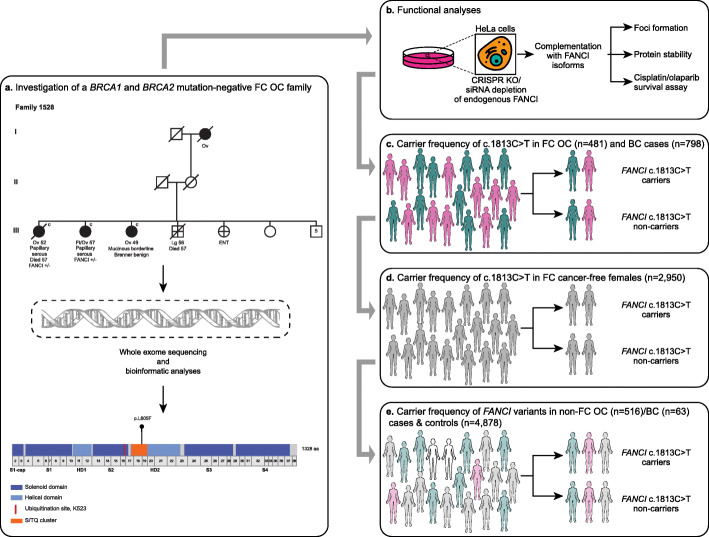
Study design for discovery and investigation of *FANCI* variants. **a** Pedigree F1528, a rare FC family with four cases of OC, in which *FANCI* c.1813C>T; p.L605F was discovered. WES was performed on the sisters, Ov 52 and FtOv 57 in generation III, who are *BRCA1* and *BRCA2* pathogenic variant negative. Cancer type (Ov: ovarian, Ft: fallopian tube, Lg: lung, and ENT: ear, nose, throat) and age of diagnosis are shown; **c** next to a symbol denotes a confirmed cancer case. The location of p.L605F is shown (bottom). Solenoid domain: antiparallel pairs of α-helices that form α-α superhelix segments; Helical domain: α-helices; Ubiquitination site, K523: site of monoubiquitination by the FA core complex to allow downstream FA pathway function [36, 37]; S/TQ cluster: location of conserved phosphorylation sites [34]. **b** Functional analyses of FANCI isoforms using HeLa cells. **c–e** Estimation of *FANCI* c.1813C>T; p.L605F carrier frequency in cases and controls. FANCI domains were adapted from pfam (https://pfam.xfam.org). *FANCI* exon locations adapted from University of California Santa Cruz Genome Browser (https://genome.ucsc.edu)

The *FANCI* c.1813C>T variant was initially discovered in family F1528 and has since been updated to include new information, including histopathology of OC and a reported case of ear, nose, and throat cancer (Fig. 1). PBL DNA (~ 500 ng) from two sisters from this family was captured with the Agilent SureSelect 50 Mb exome capture oligonucleotide library, and then sequenced with paired-end 100 bp reads on Illumina HiSeq 2000. After removing putative PCR-generated duplicate reads using Picard (V.1.48), sequencing reads were aligned to human genome assembly hg19 using a Burroughs–Wheeler algorithm (BWA V.0.5.9). Sequence variants were called using Samtools (V.0.1.17) mpileup and varFilter meeting the following criteria: at least three variant reads, ≥ 20% variant reads for each called position, and Phred-like quality scores of ≥ 20 for SNPs and ≥ 50 for small insertions or deletions. Annovar [51] and custom scripts were used to annotate variants according to the type of variant, Single Nucleotide Polymorphism database designation (dbSNP), Sorting Intolerant from Tolerant (SIFT) score [52], and allele frequency data from the 1000 Genomes Project [53] and National Heart, Lung and Blood Institute (NHLBI) Exome Sequencing Project (ESP) v.2014 [54]. Then, the variant list was organized to select top candidate variants that were shared in common among the two sisters by de-prioritizing the following: (1) synonymous or intronic variants other than those affecting the consensus splice sites; (2) variants seen in more than 5 of 416 exomes from patients with rare, monogenic diseases unrelated to cancer that were independently sequenced and available at the McGill Genome Centre (MGC); and (3) variants with a frequency ≥ 1% in either the 1000 Genomes Project or NHLBI exome datasets. Using a candidate gene approach, we then further prioritized the list of candidates based on their role in FA-HR pathways. Using this strategy, *FANCI* c.1813C>T was the only candidate remaining on the list of prioritized variants (*n* = 276) shared in common between the two sisters in family F1528. The presence of the *FANCI* variant was verified using Integrative Genomics Viewer (IGV) [55]. The *FANCI* c.1813C>T variant was validated by targeted PCR analysis and bi-directional Sanger sequencing at the MGC using standard methods (see Additional file 1: Table S3).

Since the initial discovery of the *FANCI* variant in family F1528, newer WES capture kit technology and bioinformatic tools became available, and thus we repeated our analysis with DNA from the same sisters from this family. WES and bioinformatic analyses were again performed at the MGC using Roche NimbleGen SeqCap® EZ Exome Kit v3.0 (Roche Sequencing) followed by HiSeq 100 bp paired-end sequencing (Illumina) applying the manufacturer’s protocols. Sequencing reads were aligned to human genome assembly hg19 using BWA-MEM v0.7.17, then deduplicated using Picard v2.9.0 (Broad Institute). Bases were recalibrated using the GATK best practices. Variants were called using HaplotypeCaller available from GATK v3.5 (Broad Institute) and recalibrated according to GATK best practices. The filtered variants were then annotated and loaded into a GEMINI v0.19.1 database as per the recommended workflow. Data was filtered for non-synonymous rare variants (variant allele frequency [VAF] < 1%) deduced from a publicly available database Genome Aggregation Database (gnomAD) v2.1.1 [56] identified in genes with reported function in DNA repair pathways (*n* = 276 [57]). *FANCI* c.1813C>T was once again the only variant directly involved in the FA-HR DNA repair pathway identified in both sisters. The presence of the *FANCI* variant was again confirmed by IGV [55] and validated by PCR analysis and Sanger sequencing at the MGC using standard methods (see Additional file 1: Table S3).

### Genetic analyses of candidate *FANCI* variants in FC cancer cases and cancer-free controls

In FC cancer cases, carriers of *FANCI* c.1813C>T were identified by targeted genotyping of PBL DNA samples or from surveying available WES data (subjected to the same latest WES technology and data analysis pipeline as described above) from affected cases in our study groups (see Additional file 1: Table S1). PBL DNA from OC or BC cases were genotyped using a custom TaqMan® genotyping assay [58] based on established methods (see Additional file 1: Table S4). Where PBL DNA was no longer available from the study case, genomic DNA extracted from the tumour (if available) was provided by the RRCancer biobank for genotyping. PBL DNA from sporadic BC cases were genotyped using Sequenom® iPLEX® Gold Technology at the MGC [42]. Samples that were removed from the analysis were due to poor DNA quality (*n* = 30), duplication (*n* = 1), or were from cases exceeding age limit criteria (70 years or older when diagnosed with first invasive BC; *n* = 2). Results from a total of 558 cases were evaluated for *FANCI* c.1813C>T carrier status. The *FANCI* locus (NC_000015.9: g.89828441C>T) was reviewed in WES data, validated by IGV analysis, and *FANCI* c.1813C>T variant carriers verified by Sanger sequencing as described (see Additional file 1: Table S3).

To identify carriers of *FANCI* c.1813C>T in CARTaGENE FC cancer-free controls, data was extracted from available genotyping sets derived from germline DNA of subjects that were genotyped in three different batches using two different genotyping platforms (Illumina and Affymetrix; see Additional file 1: Table S2). Data was imputed when there was no representative probe for a locus on the genotyping array using the Sanger Imputation Service with Haplotype Reference Consortium (release 1.1) as the reference panel [59]. Pre-phasing and imputation was performed using Eagle2 [60] and the positional Burrows-Wheeler transform (PBWT) [61]. Samples were removed as part of quality control to improve imputation of the array (see Additional file 1: Table S2).

Two-sided Fisher’s exact test was used to compare frequencies of *FANCI* c.1813C>T carriers in the cases and controls or between different study subjects, where a *p* value ≥ 0.05 was considered significant. Odds ratios and 95% CIs were estimated for all study subjects for this allele.

### Identification of candidate *FANCI* in various populations

Candidate *FANCI* variants were identified by investigating genotyping data available from OCAC, BCAC, and TCGA biobank resources or derived from the genetic analysis of AUS and CDN study groups (see Additional file 1: Table S1). Rare (VAF < 1%) *FANCI* variants were subjected to bioinformatic analyses using 13 in silico tools, to predict the effect of the nucleotide change(s), which includes four tools for conservation and three tools to predict splice site variants. These tools were selected for the best predictive performance [62]. Conservation tools included the following: Genomic Evolutionary Rate Profiling (GERP++) [63], Phylogenetic *P* values (PhyloP) 100 way in vertebrates [64], Phylogenetic Analysis with Space/Time models Conservation (PhastCons) 100 way in vertebrates [65], and Site-specific Phylogenetic analysis (SiPhy) 29 way in mammals [66], where variants were conserved if ≥ 2 in GERP++ and ≥ 0.4 in all other tools. In silico tools for missense variants included the following: Combined Annotation Dependent Depletion (CADD) [67] v1.6, Consensus Deleteriousness (Condel) [68], Eigen [69] v1.1, Meta-analytic Logistic Regression (MetaLR) [70], Meta-analytic Support Vector Machine (MetaSVM) [70], Variant Effect Scoring Test (VEST) [71] v4.0, and Rare Exome Variant Ensemble Learner (REVEL) [72], where variants were candidates if ≥ 15 in CADD and ≥ 0.4 in all other tools. Splice site variants were analysed with Maximum Entropy Modeling of Short Sequence Motifs (MaxEntScan) [73] (splicing change if difference ≥ |2| and Database Splicing Consensus Single Nucleotide Variant v4.0 (dbscSNV) tools, AdaBoost (ADA) and Random Forest (RF) [74] (splicing change if score ≥ 0.4). Variants were considered candidates if they were predicted to be pathogenic/deleterious in ≥ five out of seven tools and ≥ two out of four conservation tools for missense variants or all three tools for splice site variants (± 5 nucleotides from the exon-intron junction). Nonsense and frameshift variants were considered candidates, but in-frame deletions were not. Variants were annotated using the Ensembl Variant Effect Predictor [75].

### Genetic analysis of *FANCI* locus in OC and BC cases and controls from consortia databases

The *FANCI* locus was investigated in available OCAC and BCAC data. The log_2_OR, standard error (SE), *χ*^2^, and *p* value for 25,509 epithelial OC cases (22,406 invasive cancer) and 40,491 controls of EUR ancestry [44] were derived from OCAC resource. The log_2_OR, standard error (SE), *χ*^2^, and *p* value for 46,785 cases and 42,892 controls of EUR ancestry [45, 46] were derived from BCAC resource. Data was also available for carriers of *BRCA1* c.4327C>T and *rs8037137* loci, which were used as comparators (see Additional file 1: Table S5). All rare (VAF < 1%) *FANCI* variants identified in the OCAC and BCAC resource were subjected to the same bioinformatic analyses using in silico tools as described.

### Genetic analysis of *FANCI* locus in AUS HGSC cases and controls

The *FANCI* locus was investigated in germline sequencing data available from WES analysis of 516 AUS HGSC cases as previously described [47] (see Additional file 1: Table S1) and 4878 AUS controls from the lifepool study [48]. The identified rare (VAF < 1%) variants found in *FANCI* were subjected to the same bioinformatic analyses using in silico tools as described.

### Genetic analysis of *FANCI* locus in CDN BC cases

The *FANCI* locus was investigated in germline sequencing data available from other non-FC CDN study groups subjected to WES analysis of PBL DNA from subjects with OC, BC, or pancreatic cancer (*n* = 63) (Additional file 1: Table S1). *FANCI* variants were selected from PE125 WES data that was generated using the Nextera Rapid Capture Exome enrichment kit (Illumina) followed by HiSeq-4000 sequencing performed by the CRUK CI genomics core facility in the UK. Variant Call Format files were generated with a standard pipeline following GATK Best Practices recommendations for WES data. The identified rare (VAF < 1%) variants found in *FANCI* were subjected to the same bioinformatic analyses using in silico tools as described.

### Genetic analysis of *FANCI* locus in TCGA Pan-Cancer cases

Processed *FANCI* mRNA expression and clinical data from TCGA Pan-Cancer data set were downloaded from University of California Santa Cruz Xena Browser [50]. Kaplan-Meier curves for overall survival were performed for all 35 cancer types from the Pan-Cancer TCGA [76]. Samples were dichotomized into high and low *FANCI* expression groups based on the median. For OC cases, data was parsed based on *BRCA1* and *BRCA2* pathogenic variant status (germline and somatic) according to TCGA reporting of variants. WES data from 412 OC cases of the Pan-Cancer TCGA set was downloaded and annotated using wANNOVAR [51]. The identified rare (VAF < 1%) variants found in *FANCI* were subjected to the same bioinformatic analyses using in silico tools as described.

### Genetic analysis of variants in known OC-predisposing genes and DNA repair genes in FC *FANCI* c.1813C>T carriers

Rare (VAF < 1%) variants that were identified in known high-risk epithelial OC-predisposing genes in the analysis of WES data from *FANCI* c.1813C>T carriers was investigated using various bioinformatic tools. *BRCA1* and *BRCA2* variants were classified for their pathogenicity using BRCA Exchange [31] and ClinVar [77]. Rare (VAF < 1%) variants in DNA repair pathway genes (*n* = 276 [57]) were evaluated in *FANCI* c.1813C>T carriers. The only variant identified that was shared in all cases was *POLG* c.2492A>G (see Additional file 2) and it was pursued further as described below.

The allele frequency of *POLG* c.2492A>G was determined by investigating selected index OC or BC FC cases as above. Carriers of *POLG* c.2492A>G were identified by targeted genotyping of PBL DNA samples or from surveying available WES data from affected cases from our study groups as described. PBL DNA from OC or BC cases were genotyped using a custom TaqMan® genotyping assay [58] based on established methods (see Additional file 1: Table S4). *POLG* c.2492A>G was reviewed in available WES data as above. Genotyping data from CARTaGENE for cancer-free FC controls was investigated as above, including imputation (see Additional file 1: Table S2). *POLG* c.2492A>G was subjected to the same bioinformatic analyses using in silico tools as described.

### Cell lines, cell culture, and reagents

HeLa cells and OVCAR-4 cells were grown in Dulbecco’s modified Eagle’s medium (Corning™ cellgro™) and Dulbecco’s modified Eagle’s medium/Nutrient Mixture F-12 (Gibco^TM^) respectively, both supplemented with 10% foetal bovine serum (Gibco^TM^), at 37 °C, 5% CO_2_, and 20% O_2_. OVCAR-3 cells were grown in RPMI supplemented with 0.01 mg/ml bovine insulin and 20% foetal bovine serum (Gibco^TM^), at 37 °C, 5% CO_2._ HeLa cells knockout (KO) for *FANCI* were obtained using the ALT-R CRISPR-Cas9 system from Integrated DNA Technologies^TM^. Cells were transfected with crRNA:tracrRNA:Cas9 RNP-complexes (crRNA sequence: AATCCCCCGATTCCACCAAC), according to the manufacturer’s guidelines for RiboNucleoProtein transfection using RNAimax. After transfection, genomic DNA from the pool of transfected cells was extracted using QIAamp DNA Mini Kit (Qiagen, ref 51306). A 500-bp DNA region containing the sgRNA complementing sequence was amplified by PCR from 400 ng of genomic DNA with the Thermo Scientific™ Phusion™ High-Fidelity DNA Polymerase and verified by sequencing using the following primers: Forward: 5′-GTTACTGGACTTCTCAAAAGCTGTAAG-3′ and Reverse: 5′- CTAGGTTGGGCACTTAAGTTTTCCT-3′. Sequencing results from non-transfected cells and genetically altered cells were compared using TIDE software to estimate the percentage of genetically altered cells. Clones were then generated and selected based on FANCI protein depletion using western blot analysis. Two clones, clones 1 and 2, were used in this study.

When specified, cells were treated with mitomycin C (MMC) from *Streptomyces caespitosus* (Millipore-Sigma, ref M0440) or formaldehyde (BAKER ANALYZED® ACS, J.T. Baker®, ref CAJT2106). For protein stability assays, cycloheximide (CHX) (Millipore-Sigma, ref C4859) was used at a final concentration of 100 μg/ml.

### siRNA transfection and complementation assays

Approximately 2.5 × 10^5^ HeLa cells were transfected with 50 nM of siCTL (UUCGAACGUGUCACGUCAA) or siFANCI (UGGCUAAUCACCAAGCUUAA) with RNAimax (Invitrogen) according to the manufacturer’s protocol. Then, after 24 h, cells were transfected again with the same siRNAs. After 6 h, cells were complemented with the indicated pcDNA3-Flag-FANCI constructs of Flag-FANCI or pcDNA3 empty vector (EV) using Lipofectamine 2000 according to the manufacturer’s protocol, using the following quantities of plasmids: 1 μg of wild type (WT) and EV, 3 μg of p.L605F, and 1.5 μg of p.P55L. In the case of HeLa FANCI^−/−^ cells, 3.5 × 10^5^ cells were seeded and directly transfected with pcDNA3 or pEYFP-C1 constructs after 24 h. For immunofluorescence, peGFP and piRFP670-N1 plasmids, respectively, were co-transfected at a volume corresponding to 10% of the quantity of transfected pcDNA plasmid construct. Approximately 3 × 10^5^ OVCAR-3 or OVCAR-4 cells were transfected with 50 nM of siCTL or siFANCI with RNAimax (Invitrogen) according to the manufacturer’s protocol. After 24 h, cells were complemented with the indicated constructs of pcDNA3-Flag-FANCI constructs or pcDNA3 EV using Lipofectamine 3000 according to the manufacturer’s protocol, using the following quantities of plasmids: 2 μg of WT FANCI or EV, 3 μg of p.L605F.

### Protein extraction and immunoblotting

Cells were collected by trypsinization and rinsed once in cold PBS. Cell pellets were then incubated in lysis buffer (10 mM HEPES pH 7.4, 10 mM KCl, 1% Triton, 150 mM NaCl, 30 mM Na_2_P_2_O_7_.10H_2_O, 1 mM EDTA and 1 μg/ml Leupeptin, 3.4 μg/ml Aprotinin, 1% PMSF, 5 mM NaF, 1 mM Na_3_VO_4_, Complete™ EDTA-free Protease Inhibitor Cocktail (Roche)) for 30 min on ice. Cell lysates were then sonicated for 5 min (30 s on, 30 s off, high, Bioruptor) and centrifuged for 30 min, 13,000 rpm, 4 °C. Supernatant was then processed for immunoblotting analysis using the indicated antibodies.

### Antibodies for western blotting and immunofluorescence assays

The antibodies used were anti-FANCI (A7) (Santa Cruz Biotechnology, ref sc-271316, 1:100 for western blot), anti-FANCD2 (Novus, ref NB100-182D1, 1:5000 for western blot, 1:1000 for immunofluorescence), anti-Flag (Cell signaling Technologies, ref 8146, 1:1600 for immunofluorescence), and anti-vinculin (Sigma, ref V9131, 1:100,000 for western blot). Horseradish peroxidase-conjugated anti-rabbit IgG or anti-mouse (1:10,000; Jackson ImmunoResearch) were used as secondary antibodies for western blot. For immunofluorescence, Alexa Fluor secondary antibodies from Life Technologies (Goat anti-mouse Alexa fluor 568 A-11004, Goat anti-rabbit Alexa fluor 568 A-11011, Goat anti-rabbit Alexa fluor 488 A-11008) were used at a 1:1000 dilution.

### Cisplatin and olaparib cell survival assays

Approximately 3 × 10^5^ HeLa FANCI^−/−^ cells were seeded into one well of a six-well plate. After 24 h, cells were complemented with the indicated Flag-FANCI construct using Lipofectamine 2000 (Invitrogen), and then after another 24 h seeded in triplicate into a Corning 3603 black-sided clear bottom 96-well microplate at a density of 3500 cells per well. The remaining cells were stored at − 80 °C until processing for protein extraction and immunoblotting as described above. Once attached to the plate, the cells were exposed to different concentrations of either 0–300 nM cisplatin (Tocris, #2251) or 0–2.5 μM olaparib. After 3 days of treatment, nuclei were stained with Hoechst 33342 (Invitrogen) at 10 μg/ml in media for 45 min at 37 °C. Images of entire wells were captured at × 4 magnification using a Cytation^TM^ 5 Cell Imaging Multi-Mode Reader and Hoechst-stained nuclei were quantified with the Gen5 Data Analysis Software v3.03 (BioTek Instruments). Cell viability was expressed as percentage of cell survival in cisplatin or olaparib-treated cells relative to vehicle (DMSO)-treated cells. Results represent the mean ± standard error of the mean (SEM) of at least three independent biological replicates, each performed in technical triplicate.

### Protein stability assays

To test the stability of Flag-FANCI variants, HeLa FANCI^+/+^ cells were first transfected with siRNA targeting FANCI and then complemented with Flag-FANCI constructs as described above. For HeLa FANCI^−/−^ clones, and OVCAR-3 or OVCAR-4, cells were directly transfected with Flag-FANCI constructs. Twenty-four hours after DNA transfection, cells were seeded in 6-well plates at 5 × 10^5^ cells/well for HeLa and 3.5 × 10^5^ cells/well for OVCAR-3 or OVCAR-4 and grown overnight. Cells were then treated with CHX (100 μg/ml) and MMC (50 ng/ml) or formaldehyde (300 μM) or no genotoxic treatment for the indicated times. At each time point (t0, t1.5h, t3h, t4h, t5h, t6h, and t8h), cells were collected by trypsinization and snap-frozen after a wash in cold PBS. Samples were prepared for immunoblotting as described above. A first western blot was performed with all t0 timepoints to adjust quantity of samples to load for the whole kinetic in order to have comparable amounts of Flag-FANCI constructs at t0. Flag-FANCI WT and Flag-FANCI p.L605F were run on the same gel.

### Immunofluorescence analyses

HeLa FANCI^−/−^ cells were complemented with either FLAG-FANCI variants (1 μg of WT, 3 μg of p.L605F, and 1.5 μg of p.P55L) and 0.1 μg of transfection control peGFP to identify transfected cells, or pEYFP-C1-FANCI (1 μg of WT, 3 μg of p.L605F) and 0.1 μg of transfection control piRFP670-N1 to identify transfected cells. One microgram of pcDNA3 or pEYFP-C1 was used as EV. After 18 h, cells were seeded on a glass coverslip for 8 h and then treated with 50 ng/ml MMC for 18 h and processed for immunofluorescence with anti-FANCD2 (Novus, ref NB100-182D1, 1:1000) antibody according to the protocol provided by Cell Signaling Technologies for Flag antibody (ref 8146). Briefly, cells were fixed in PBS-PFA 4% for 15 min at room temperature and blocked and permeabilized in Blocking Buffer (1× PBS / 5% normal serum / 0.3% Triton™ X-100) for 30 min at room temperature. Incubation with anti-FANCD2 antibody, diluted in Antibody Dilution Buffer: (1X PBS / 1% BSA / 0.3% Triton™ X-100), was performed for 2 h, room temperature. After three washes of 5 min in PBS, Alexa Fluor secondary antibody from Life Technologies (Goat anti-rabbit Alexa fluor 568 A-11011) was used at 1:1000 in antibody dilution buffer and incubated for 1 h at room temperature. Finally, slides were incubated in DAPI for 15 min and washed two more times in PBS for 5 min, and ProLong® Gold Antifade Mountant (Invitrogen^TM^) was used as mounting medium. FANCD2 and YFP-FANCI foci were counted in transfected cells according to the transfection control used (peGFP- or iRFP-positive cells). HeLa FANCI^+/+^ cells were transfected with siRNA and complemented with siRNA-resistant FANCI variants or EV as described above. After 18 h, cells were seeded on a glass coverslip for 8 h and then treated with 50 ng/ml MMC for 18 h and processed for immunofluorescence with anti-Flag (Cell signaling Technologies, ref 8146, 1:1600) and anti-FANCD2 (Novus, ref NB100-182D1, 1:1000) as described before except that incubation with primary antibody was performed at 4 °C, overnight in a humid chamber. Alexa Fluor secondary antibodies from Life Technologies (ref A-11008, A-11004) were used at 1:1000. In HeLa FANCI^−/−^ cells, FANCD2 foci were counted in GFP-positive cells. In that case, only Flag-positive cells were taken into consideration for the quantification of FANCD2 foci. Each dot represents a nucleus and the red line corresponds to the mean of FANCD2 or FANCI foci per nucleus and error bars the SEM. Statistical analysis was performed using GraphPad Prism 8 (Kruskal-Wallis test).

### Anti-Flag pulldown assays

After siRNA transfection and complementation with Flag-FANCI WT or p.L605F, HeLa FANCI^+/+^ cells were lysed in lysis buffer (50 mM Tris–HCl, pH 7.5, 150 mM NaCl, 0.5% NP-40) containing protease and phosphatase inhibitors (1 mM PMSF, 0.019TI U/ml aprotinin, 1 mg/ml leupeptin, 5 mM NaF, and 1 mM Na_3_VO_4_) incubated for 30 min on ice, and lysed by sonication. Insoluble material was removed by high-speed centrifugation (13,000 rpm at 4 °C) and each immunoprecipitation was carried out using soluble protein extract in 1 ml of lysis buffer. Fifty millilitres of anti-Flag M2 affinity gel (Sigma) and 70 U of DNase I were added and incubated at 4 °C for 2.5 h. Beads were washed three times with washing buffer (50 mM Tris–HCl, pH 7.5, 250 mM NaCl, 0.5% NP-40), and proteins were eluted with 60 μl of Laemmli buffer. Proteins were visualized by western blotting using the appropriate antibodies. Flag-FANCI p.L605F variant immunoprecipitation was overloaded in order to have the same amount of protein immunoprecipitated as in the Flag-FANCI WT lane and be able to compare co-immunoprecipitated FANCD2. Experiment has been performed twice.

### FANCI protein expression by immunohistochemistry (IHC) analysis of HGSC tumours and normal tissues

Slides containing 4 micron slices of TMAs containing 0.6 mm FFPE tissue cores (spaced 0.2 mm apart) of HGSC (*n* = 101) [78] and normal fallopian tube (*n* = 15) tissues, and *FANCI* c.1813C>T carrier tumour tissues (*n* = 8) were stained using the BenchMark XT automated stainer (Ventana Medical System Inc., Roche). Antigen retrieval was carried out with Cell Conditioning 1 solution for 1 h. The FANCI polyclonal antibody (Sigma HPA039972 dilution 1/200) was automatically dispensed and the TMAs were incubated at 37 °C for 1 h. The Ultra View DAB detection kit was used, and the slide was counterstained with haematoxylin. The TMAs were scanned with a 20 × 0.75 NA objective by VS-110 Olympus.

Staining patterns were evaluated by two independent observers. Intensity of staining was scored for all cores using a 4-point system; zero referring to no detectable staining to three referring to the highest staining intensity. As each sample was present in the TMA in duplicate, each case received four scores (two for the first core and two for the second core). The mode score was used for analysis where possible; otherwise, the average score was used. The interobserver correlation for IHC analysis of the TMA of HGSC samples was 89%. Staining patterns and analyses from the TMA containing HGSC samples and normal fallopian tube samples were evaluated without prior knowledge of carrier status for *FANCI* c.1813C>T. All HGSC and normal fallopian tube samples were genotyped for *FANCI* c.1813C>T variant as described, and one previously known carrier was identified (PT0004). Samples that could not be scored were removed from further analysis (*n* = 7 HGSC samples, *n* = 2 fallopian tube epithelium [FTE] samples). A second TMA that contained 10 samples from eight *FANCI* c.1813C>T carriers (in duplicate) were also scored separately: the results from one sample from this TMA was removed from analysis due to poor tissue quality.

Spearman correlation was used to measure the strength of the correlation of staining intensity and survival data with clinical data as continuous variables. Survival curve was calculated according to Kaplan-Meier method coupled with a log rank test. Univariable Cox hazard models were used to estimate the hazard ratio as categorical data. All statistical analysis was done using Statistical Package for the Social Sciences software version 24 (SPSS, Inc) and results deemed statistically significance at *p ≤* 0.05.

## Results

### Discovery of *FANCI* c.1813C>T as a candidate

We previously reported a rare *BRCA1* and *BRCA2* pathogenic variant negative OC family (F1528) in a study of the histopathology of OC and *BRCA1* and *BRCA2* pathogenic variant carrier status of FC cancer families [79]. To clarify, *BRCA1* and *BRCA2* pathogenic variants were not identified in either sibling using two different WES platforms, which is consistent with independent clinical genetic testing results. To investigate if other candidate variants could be contributing to cancer risk in this family, WES and bioinformatic analyses were performed on PBL DNA available from two affected siblings both of whom had HGSC [79]. We selected rare (VAF < 1%) variants (*n* = 276) as candidates that were inherited in the heterozygous state and shared in common with the affected sisters. The only DNA repair pathway gene identified with a variant was *FANCI* (c.1813C>T; p.L605F). This was an intriguing candidate to investigate given that family F1528 is predicted to harbour a pathogenic variant in *BRCA1* or *BRCA2* (Manchester score [80, 81]: *BRCA1* = 29, *BRCA2* = 20). As *FANCI* plays a role in FA-HR pathway it may be associated with phenotypically similar cancer families that have implicated *BRCA1* and *BRCA2* [79, 82] (Fig. 1). Preliminary in silico tools predicted this variant, located within the S/TQ phosphorylation cluster [34] of the encoded protein, to be highly conserved and probably damaging. However, at the time of discovery, the overall allele frequency of *FANCI* c.1813C>T from available databases was 0.76% in the NHLBI ESP v.2014 [54] and 0.2% in the 1000 Genomes Project [53]. These allele frequencies were notably higher than expected for individual pathogenic variants found in known OC-predisposing genes, such as *BRCA1* and *BRCA2* (0.001%). Therefore, we performed a molecular investigation before pursuing extensive genetic analyses of our study groups.

### In cellulo and in vitro analysis revealed FANCI p.L605F isoform behaves differently than WT protein

**Fig. 2:**
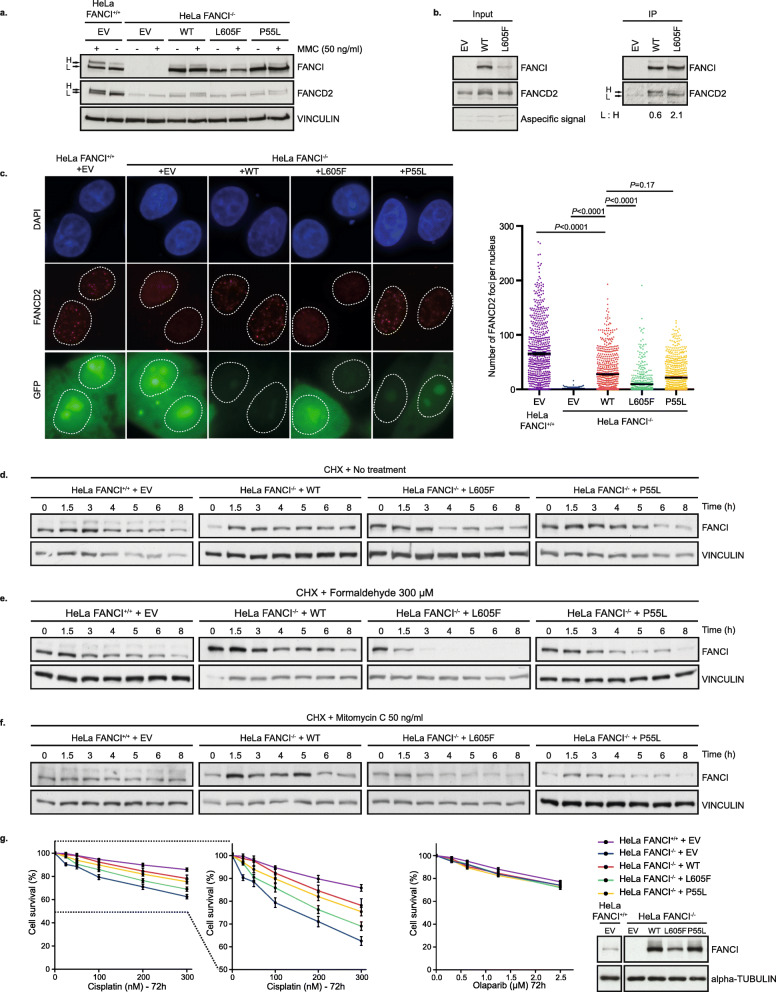
The isoform with the p.L605F variant impairs FANCI stability and function. **a** Western blots of HeLa cells with the *FANCI* gene (FANCI^+/+^) or with the *FANCI* gene knocked out (FANCI^−/−^). HeLa FANCI^−/−^ cells from clone 1 were complemented with constructs of Flag-FANCI wild type (WT), p.L605F or p.P55L, or an empty vector (EV) and treated with 50 ng/ml MMC for 18 h. The upper band, H, shows the ubiquitination of FANCI and FANCD2 after treatment. The lower band, L, corresponds to non-ubiquitinated FANCI or FANCD2. VINCULIN was used as a loading control. Experiment was repeated three times. **b** HeLa FANCI^+/+^ cells were transfected with siRNA targeting FANCI and then complemented with Flag-FANCI siRNA-resistant constructs or an EV. Cells were treated with 50 ng/ml MMC for 18 h followed by FLAG immunoprecipitation. The left panel shows FANCI constructs expression and the right panel the immunoprecipitated fractions. The p.L605F immunoprecipitation fraction sample was super-loaded to have the same signal after FANCI WT complementation. The ratio between the upper band (H) and lower band (L) for the immunoprecipitated FANCD2 is shown. **c** Immunofluorescence of HeLa FANCI^−/−^ cells from clone 1 that were complemented with constructs of Flag-FANCI and 0.1 μg of empty GFP vector was used as a transfection control. The adjacent scatter plot shows the number of FANCD2 foci in GFP-positive cells after treatment with MMC (50 ng/ml, 18 h). Mean with SEM is represented. The Kruskal-Wallis test was used to compare groups and the *P* value is shown for each test. Experiment has been performed in triplicate. **d–f** Western blot analysis of HeLa FANCI^−/−^ cells from clone 1 that were complemented with constructs of Flag-FANCI and treated with cycloheximide (CHX) and either mock-treated (**d**) or treated with damaging agents formaldehyde (**e**) or MMC (**f**) for different lengths of time at the indicated concentrations. At each time point, whole cell extracts were analysed by western blot to assess protein levels. Experiment has been done in triplicate. **g** Survival curves of HeLa FANCI^−/−^ cells from clone 1 that were transfected with the different constructs of Flag-FANCI. Cell viability was monitored following cisplatin or olaparib treatments for 72 h and was assessed by counting remaining nuclei. Curves represent mean with SEM of three biological replicates. Western blots were used to monitor expression and shown here as an example. Alpha-tubulin was used as a loading control. Full blots are shown in Additional file 4

FANCI belongs to the FA-HR DNA repair pathway that has been mainly described to be involved in ICL repair induced by DNA cross-linking agents, such as MMC. Briefly, when DNA replication forks are blocked by the presence of an ICL, FANCM recognizes the lesion, recruits the FA core complex which will ubiquitinate the heterodimer FANCI-FANCD2. Essential to downstream FA pathway function is this interdependent ubiquitination of both FANCI and FANCD2 [36–38], leading to DNA repair through DNA lesion excision of the DNA crosslink, DNA translesion synthesis, and HR. The functionality of this pathway can be assessed in cellulo by monitoring the ubiquitination of FANCI and FANCD2 after MMC treatment. To investigate the functional impact of FANCI p.L605F isoform, both HeLa CRISPR FANCI KO (Fig. 2a–g, Additional file 3: Fig. S1, Additional file 4) or HeLa FANCI siRNA knockdown (KD) cells (Additional file 3: Fig. S1, Additional file 4) were complemented with the FANCI p.L605F isoform and treated with MMC. Western blot analysis first showed decreased levels of FANCI p.L605F isoform, unlike the FANCI p.P55L isoform encoded by variant c.164C>T which has been reported to exhibit WT function [37] (Fig. 2a, Additional file 3: Fig. S1, Additional file 4). Increasing the quantity of transfected *FANCI* c.1813C>T DNA by threefold did not overtly increase the level of protein expression comparable to that seen in the WT FANCI or p.P55L isoform (Additional file 3: Fig. S1, Additional file 4). We then looked at the impact of MMC treatment on FANCI and FANCD2 ubiquitination depending on FANCI status (Fig. 2a and Additional file 3: Fig. S1, Additional file 4). In WT or siCTL cells, both proteins are modified, as shown by the presence of the upper band (H). As expected, in the absence of FANCI, FANCD2 ubiquitination is lost. Complementation with WT FANCI or FANCI p.P55L isoform partially rescued the phenotype, though rescue was less evident in cells complemented with FANCI p.L605F. To confirm this, we then looked at FANCD2 ubiquitination after immunoprecipitation of FANCI WT or FANCI p.L605F isoform in presence of MMC. Given that the level of FANCI p.L605F isoform is lower than the WT in the input (Fig. 2b, left panel, Additional file 4), we overloaded the immunoprecipitated fraction of the variant in order to have the same signal in both lanes to be able to compare the two conditions. Though FANCI p.L605F isoform co-immunoprecipitates with FANCD2, ubiquitination levels of FANCD2 were severely diminished as compared to those in FANCI WT expressing cells (Fig. 2b, right panel, Additional file 4) confirming our results (Fig. 2a, Additional file 4). These observations suggest that while physical interactions between FANCI p.L605F isoform and FANCD2 proteins are maintained the altered FANCI isoform may affect ubiquitination of FANCD2. As ubiquitination of FANCD2 is required to form MMC-induced foci, we then looked at FANCD2 foci formation in both KO and KD cells. Consistent with this role, the expression of FANCI p.L605F led to a significant reduction in the number of FANCD2 foci in transfected cells, while both WT and FANCI p.P55L isoforms were able to rescue the loss of FANCD2 foci observed in absence of FANCI (Fig. 2c, Additional file 3: Fig. S1, Additional file 4). Moreover, a concomitant reduction of GFP-FANCI p.L605F was also observed (Additional file 3: Fig. S2, Additional file 4).

As the expression of FANCI p.L605F appeared to be lower than the WT or p.P55L isoforms, even when increasing the quantity of plasmid (Fig. 2a, Additional file 3: Fig. S1, Additional file 4), we suspected that this protein isoform was unstable. Upon MMC or formaldehyde treatment, both known to induce DNA damage repaired by the FA-HR pathway, cells expressing FANCI WT protein, or either of the p.L605F and p.P55L isoforms, were treated with CHX to inhibit protein synthesis. FANCI protein levels decreased over time in response to both DNA damaging agents (Fig. 2d–f, Additional file 3: Fig. S1, Additional file 4). We recapitulated our findings in OVCAR-3 and OVCAR-4 cell lines to determine if FANCI is also unstable in an OC cell line background (Additional file 3: Fig. S2, Additional file 4). The effect was more prominent in FANCI p.L605F expressing cells as compared to WT FANCI or p.P55L expressing cells. These observations suggest that treatment with genotoxic agents exacerbates FANCI p.L605F protein instability, as it has been previously described for BRCA2 protein [83]. This is in agreement with the observation that FANCI p.L605F failed to complement survival of the HeLa FANCI^−/−^ cells that were challenged with the platinum compound cisplatin (Fig. 2g, Additional file 3: Fig. S1, Additional file 4), a drug known to induce DNA crosslinks. In contrast, albeit in accordance with the literature [84], FANCI^−/−^ cells were not sensitive to olaparib, a PARPi (Fig. 2g, Additional file 3: Fig. S1, Additional file 4).

### *FANCI* c.1813C>T carriers are enriched in familial OC cases of FC ancestry

With these promising results in hand, we assessed *FANCI* c.1813C>T carrier frequency in available PBL DNA from index OC or BC cases of FC ancestry to determine if this variant plays a role in conferring risk in phenotypically defined cancer families [17–20, 22, 23, 30, 32, 33, 40, 41, 79, 85–90]. These OC or BC cases were selected based on their family history of OC or BC, or regardless of cancer family history (sporadic cases), where *BRCA1* and *BRCA2* pathogenic variant carrier status was known [30, 32, 33, 39–41, 85, 86, 91–95]. Index OC cases from OC families (3/42, 7%) had a higher carrier frequency of *FANCI* c.1813C>T than sporadic OC cases (7/439, 1.6%, *P* = 0.048, Fisher’s exact) and sporadic BC cases (8/558, 1.4%, *P* = 0.035, Fisher’s exact). Index OC cases from OC families (3/42, 7%) and index BC cases from HBOC families (3/82, 3.7%) had a higher carrier frequency than BC cases from HBC families (3/158, 1.9%), though these differences were not statistically significant (*P* = 0.11 and *P* = 41, respectively, Fisher’s exact) (Table 1, Additional file 3: Fig. S3). When including the discovery OC family, there was an increased carrier frequency of c.1813C>T in *BRCA1* and *BRCA2* pathogenic variant negative OC families versus sporadic OC cases (*P* = 0.01, Fisher’s exact) and cancer-free females (3/23, 13%; OR = 5.8; 95%CI = 1.7-19; *P* = 0.005).

**Table 1:** Comparison of *FANCI* c.1813C>T carrier frequencies in cancer cases with French Canadian cancer-free females. All odds ratios are calculated comparing to cancer-free females

Study group^**1**^	***BRCA1 and BRCA2*** mutation status^**1**^	Case tested	Number of subjects	Number of c.1813C>T carriers (%)	OR	95% CI	***P***
OC families^2^	All	OC	42	3 (7.1)	3	0.9–9.9	0.073
	Negative		22	2^3^ (9.1)	3.9	0.89–17	0.071
	*BRCA1* positive		14	1 (7.1)	3	0.39–23	0.29
	*BRCA2* positive		6	0	NA	NA	NA
Sporadic OC cases	All	OC	439	7 (1.6)	0.63	0.29–1.4	0.25
	Negative		400	7 (1.8)	0.69	0.32–1.5	0.36
	*BRCA1* positive		18	0	NA	NA	NA
	*BRCA2* positive		21	0	NA	NA	NA
HGSC cases	All	OC	341	7 (2.1)	0.81	0.37–1.8	0.61
	Negative		310	7 (2.3)	0.9	0.41–2	0.79
	*BRCA1* positive		15	0	NA	NA	NA
	BRCA2 positive		16	0	NA	NA	NA
HBOC^2^	All	BC	82	3 (3.7)	1.5	0.46–4.8	0.52
	Negative		34	2 (5.9)	2.4	0.57–10	0.23
	*BRCA1* positive		29	0	NA	NA	NA
	*BRCA2* positive		21	1 (4.8)	1.9	0.26–15	0.52
HBC	All	BC	158	3 (1.9)	0.75	0.23–2.4	0.63
	Negative		93	2 (2.2)	0.85	0.21–3.5	0.83
	*BRCA1* positive		20	1 (5)	2.1	0.27–15	0.49
	*BRCA2* positive		45	0	NA	NA	NA
Sporadic BC cases	All	BC	558	8 (1.4)	0.57	0.27–1.2	0.13
	Negative		538	8 (1.5)	0.59	0.28 –1.2	0.16
	*BRCA1* positive		4	0	NA	NA	NA
	*BRCA2* positive		17	0	NA	NA	NA
Cancer-free females	NA	NA	2950	74 (2.5)	1		

^1^See Additional file 1: Table S1 for details of study groups

^2^There is overlap of some families but individuals were counted only once

^3^Inclusion of the discovery family (F1528) leads to 3 *FANCI* c.1813C>T carrier families out of 23 *BRCA1* and *BRCA2* pathogenic variant negative (13%; OR = 5.8; 95%CI = 1.7–20.; *P* = 0.005)

*NA* not available

### Cancer-free FC *FANCI* c.1813C>T carriers are significantly correlated with having a first-degree relative with OC

Recently, new data has become available from the CARTaGENE biobank enabling the evaluation of allele frequencies in study subjects from a cancer-free female FC population, and thus providing a more comparable reference group to our FC cancer subjects [43]. Using data from three different genotyping platforms, we estimated a 1.3% VAF in cancer-free FC females (Additional file 1: Table S6). This is not significantly different from the 1% estimated VAF in non-Finnish EURs, a population most likely to share common ancestry with FCs (France) [25, 26], as reported in the gnomAD [96] (Additional file 1: Table S6). In this database, the estimated VAF was 0.67% for the total of all study populations and varied across populations: highest in Estonians (2.1%) to none in East Asians. Rare homozygous carriers (17/134,154, 0.013%) were also identified in gnomAD. This finding did not dissuade us from pursuing this candidate variant as the in cellulo findings suggest that it may behave as a hypomorph.

The estimated carrier frequency at 2.5% in cancer-free FC females was lower than that observed in index cancer cases from OC (7.1%) and HBOC (3.7%) families, but higher than observed in sporadic OC cases (1.6%) and index BC cases from HBC families (1.9%), though these differences were not statistically significant (Table 1). Additional information was available from the CARTaGENE subjects to investigate *FANCI* c.1813C>T carrier frequency in the context of cancer family history (first-degree only), reproductive history, oral contraceptive pill use, oophorectomy, and fallopian tube ligation; all of which are host factors that are known to significantly impact lifetime risk of OC [97, 98]. We observed that cancer-free carriers (female/male) were significantly correlated with having a first-degree relative with OC (Spearman correlation = 0.037; *P* = 0.011) compared to non-carriers, when analysing data from subjects genotyped with arrays that included probes for the variant allele (*n* = 4645) (Additional file 1: Table S7). The correlation is still significant, though slightly weaker, when adding data from cancer-free subjects where genotypes were imputed (*n* = 604; Spearman correlation = 0.027; *P* = 0.047) (Additional file 1: Table S2 and S7). No other cancer type was significantly correlated with carrier status. The majority of cancer-free FC females were parous (78%, 2315/2950) and had experienced oral contraceptive pill use, oophorectomy, and/or tubal ligation (91.8%, 2710/2950) (Additional file 1: Table S8). Only 8.1% (6/74) of c.1813C>T carriers reported no risk-reducing host factors.

### Other candidate *FANCI* variants are rare in OC cases of FC ancestry

**Fig. 3:**
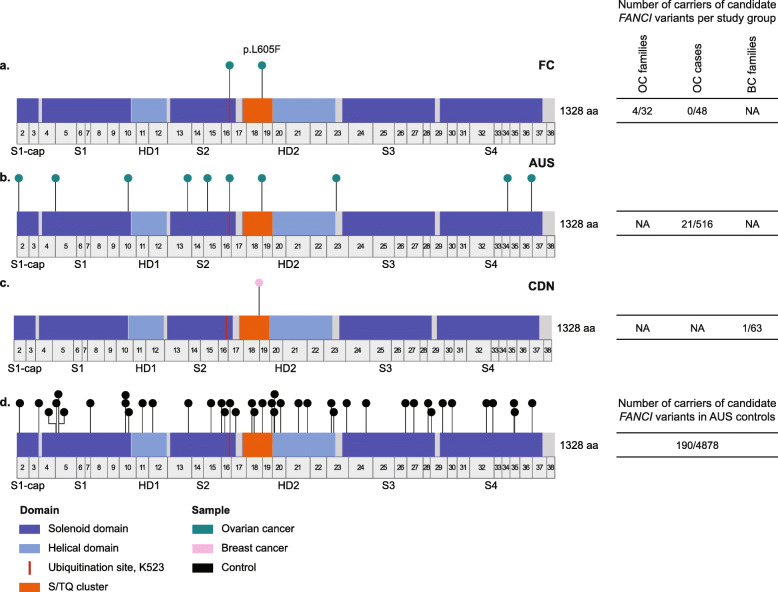
Schemata of the FANCI gene showing the location of candidate rare variants (< 1%) found in OC and/or BC in **a** French Canadian cases, **b** Australian cases, **c** Canadian non-French Canadian cases, and in **d** Australian controls. Refer to Supplementary Table 1 for study group descriptions. FANCI domains were adapted from pfam (https://pfam.xfam.org). *FANCI* exon locations adapted from University of California Santa Cruz Genome Browser (https://genome.ucsc.edu)

To determine if there are other *FANCI* variants (VAF < 1%) in FCs, we investigated available WES data from 80 familial and/or young age of onset OC cases, regardless of *BRCA1* or *BRCA2* pathogenic variant carrier status (Additional file 1: Table S1). We identified seven rare variants among 32 index OC familial cases, where one carrier was heterozygous for *FANCI* c.1573A>G; p.M525V (Additional file 1: Table S9). Although this missense variant is predicted to be highly conserved (all four conservation tools used) and damaging by in silico tools, in cellulo analyses suggested that it does not encode an aberrantly functioning protein (data not shown). Thus, *FANCI* c.1813C>T is the only plausible candidate variant identified in *FANCI* in FC OC cases (Fig. 3a).

### Co-occurrence of other candidate variants in OC-predisposing genes in *FANCI* c.1813C>T carriers

We analysed WES data from *FANCI* c.1813C>T OC (*n* = 12) carriers for the co-occurrence of pathogenic variants in known high-risk OC-predisposing genes [99]. No additional carriers of *BRCA1*, *BRCA2, BRIP1, RAD51C,* and *RAD51D* pathogenic variants were found in our familial cases. None of the sporadic OC cases (*n* = 7) carried pathogenic variants in *BRCA1, BRCA2, BRIP1, RAD51C,* and *RAD51D*. Moreover, the *FANCI* c.1813C>T variant did not co-occur in carriers of recurrent *BRCA1* [39], *BRCA2* [39], and *RAD51D* [33] in the FC population.

### OC and BC cases of non-FC ancestry also carry candidate *FANCI* variants

We identified 99 unique *FANCI* variants (VAF < 1%) in 516 AUS HGSC *BRCA1* and *BRCA2* pathogenic variant negative cases [47] and 4878 AUS cancer-free controls from available WES data (Additional file 1: Table S1). Based on in silico tools, there were 10 candidate missense variants in 516 HGSC cases (4.1%), where 10 (1.9%) cases carried *FANCI* c.1813C>T and 11 (2.1%) cases carried other variants (Table 2, Fig. 3b, Additional file 1: Table S9). We identified 42 different candidate missense variants in 190/4878 (3.9%) AUS controls, where 95 (1.9%) carried *FANCI* c.1813C>T (Fig. 3d). The number of carriers of candidate variants in *FANCI* was not significantly different between AUS cases and controls (*P* = 0.48), including for *FANCI* c.1813C>T alone (*P* = 0.81). There was no significant difference in allele frequencies of *FANCI* variants in AUS cases compared to AUS controls (Additional file 1: Table S10), though for five of eight rarest candidate *FANCI* variants (VAF < 0.1%) odds ratios were > 12 when compared to gnomAD cancer-free controls (Table 3). In contrast, *FANCI* c.1813C>T was the only variant identified in 1/63 (1.6%) familial CDN *BRCA1* and *BRCA2* pathogenic variant negative BC cases (Fig. 3c, Additional file 1: Table S9) and the carrier was known to be of Greek Canadian origin.

**Table 2:** Frequencies of carriers of candidate *FANCI* variants identified in Australian HGSC cases and controls

					Number of variant carriers^**3**^ (%)							
Study group^**1**^	Number of subjects (%)	c.13A>G p.I5V	c.286G>A p.E96K	c.824 T>C p.I275T	c.1264G>A p.G422R	c.1412C>G p.P471R	c.1573A>G p.M525V	c.1813C>T p.L605F^**4**^	c.2366C>T p.A789V^**4**^	c.3635T>C p.F1212S	c.3812C>T p.S1271F	Total number of carriers^**4**^ (%)
HGSC	516 (100)	1 (0.2)	1 (0.2)	1 (0.2)	2 (0.4)	1 (0.2)	3 (0.6)	10 (1.9)	1 (0.2)	1 (0.2)	1 (0.2)	21 (4.1)
Controls	4878 (100)	0	5 (0.1)	5 (0.1)	7 (0.1)	0	43 (0.88)	95 (1.9)	0	0	1 (0.02)	156 (3.2)
Family history of HGSC cases^2^												
≥ 2 OC cases (no BC)	7 (1)	0	0	0	0	0	1 [14]	0	0	0	0	1 (14)
1 OC case (no BC)	49 (10)	0	0	0	0	0	0	0	0	0	0	0
≥ 2 OC case and BC cases	42 (8)	0	0	0	1 (2.4)	1 (2.4)	0	5 [12]	1 (2.4)	0	0	7 (17)
**Total with OC family history**	**98 (19)**	**0**	**0**	**0**	**1 (1)**	**1 (1)**	**1 (1)**	**5 (5.1)**	**1 (1)**	**0**	**0**	**8 (8.2)**
≥ 2 BC cases (no OC)	45 (9)	0	0	0	0	0	1 (2.2)	0	0	0	0	1 (2.2)
1 BC case (no OC)	125 (24)	0	1 (0.8)	1 (0.8)	0	0	0	1 (0.8)	0	0	0	3 (2.4)
No OC or BC	248 (48)	1 (0.4)	0	0	1 (0.4)	0	1 (0.4)	4 (1.6)	0	1 (0.4)	1 (0.4)	9 (3.6)
**Total isolated HGSC**	**418 (81)**	**1 (0.2)**	**1 (0.2)**	**1 (0.2)**	**1 (0.2)**	**0**	**2 (0.5)**	**5 (1.2)**		**1 (0.2)**	**1 (0.2)**	**13 (3.1)**

^1^See Additional file 1: Table S1 for description of study groups; 95% of the participants were of Western European ancestry

^2^First-, second-, or third-degree relatives reported for OC; first- and second-degree relatives only reported for BC

^3^See Additional file 1: Table S9 for more information on *FANCI* variants found in these study groups

^4^One HGSC case carried two *FANCI* variants: c.1813C>T; p.L605F and c.2366C>T; p.A789V (see Additional file 1: Table S9)

**Table 3:** Summary statistics for candidate *FANCI* variants in the AUS population as compared to cancer-free samples from gnomAD

			Non-Finnish Europeans			All populations		
Coding DNA reference sequence^**1**^	Amino acid change	dbSNP designation	OR	95% CI	***P***	OR	95% CI	***P***
c.13A>G	p.I5V	rs200186938	NA	NA	NA	26.05	3.3– 204	1.9 × 10^−3^
c.286G>A	p.E96K	rs149243307	3.7	0.5– 27.1	0.2	0.57	0.08–4.1	0.58
c.824 T>C	p.I275T	rs142906652	1.77	0.3– 12.7	0.57	0.92	0.1–6.6	0.93
c.1264G>A	p.G422R	rs146040966	8.51	2– 35.9	3.5 × 10^−3^	17.39	4.2– 73	1 × 10^−4^
c.1412C>G	p.P471R	rs139072231	8.19	1.1– 62.4	0.04	17.36	2.3– 132	5.8 × 10^−3^
c.1573A>G	p.M525V	rs144908351	0.75	0.2–2.3	0.62	1.31	0.4–4.1	0.64
c.1813C>T	p.L605F	rs117125761	0.98	0.5–1.8	0.94	1.47	0.8–2.8	0.23
c.2366C>T	p.A789V	rs925359228	NA	NA	NA	NA	NA	NA
c.3635 T>C	p.F1212S	rs775483853	57.4	5.2– 634	1 × 10^−3^	130.26	11.8– 1439	1 × 10^−4^
c.3812C>T	p.S1271F	rs202066338	19.9	2.3– 171	6.3 × 10^−3^	12.77	1.7– 95.9	0.013

^1^ Human GRCh37/hg19

*NA* data not available for the controls

With respect to family history of cancer in AUS cases, five of the c.1813C>T carriers had a family history of OC within third-degree relatives (5/98, 5.1%), which was significantly higher than the carrier frequency of this variant in combined isolated cases of HGSC cases (5/418, 1.2%; *P* = 0.025) (Table 2). In contrast, there was no significant difference in the carrier frequency of *FANCI* c.1813C>T in combined cases with a reported family history of OC and/or BC (6/262, 2.3%) than those without (4/254, 1.6%; *P* = 0.75). *FANCI* c.1813C>T co-occurred with another candidate missense variant, *FANCI* c.2366C>T; p.A789V, in a HGSC case diagnosed at 75 years with a family history of OC. Three carriers of other candidate variants in *FANCI* (c.1573A>G; p.M525V, c.1264G>A; p.G422R, and c.1412C>G; p.P471R), with an average age of diagnosis of 52 years, also had a family history of OC (Table 2), where the carrier of p.M525V had no family history of BC. The number of carriers of candidate *FANCI* variants with a family history of OC (8/98, 8.2%) was significantly higher than isolated cases of HGSC (*P* = 0.04), but there was no significant difference when accounting for family history of OC and/or BC (*P* = 0.66).

We investigated rare variants (VAF < 1%) from imputed SNP array data that was available from two case-control studies: OCAC [44] and BCAC [45, 46]. In all, nine missense and one splice site variant were identified in OCAC and BCAC databases. *FANCI* c.1813C>T and c.824T>C were the only candidate missense variants, but the splicing variant c.3007-1G>A may be a candidate (Additional file 1: Table S11). The data imputed from the OCAC database [44] revealed that the OR for *FANCI* c.1813C>T and c.824T>C was highest in HGSC and endometrioid subtypes compared to all histopathological subtypes combined, though there was no significant difference in allele frequency in OC cases compared to controls (Additional file 1: Table S5). To compare to a known OC pathogenic variant, OCAC data was investigated for the most common pathogenic *BRCA1* variant reported in FCs, c.4327C>T; p.R1443X, and also found repeatedly in populations of EUR ancestry [39]. As similarly observed with *FANCI* c.1813C>T, the OR was highest in HGSC cases, though there was no significant difference in allele frequency when all OC subtype cases were compared to controls (Additional file 1: Table S5). There was no significant difference in allele frequency between BC cases and controls for *FANCI* c.1813C>T in a similar analysis of BCAC case-control data [45, 46] (*BRCA1* c.4327C>T; p.R1443X and *FANCI* c.824T>C data was not available in the BCAC database) (Additional file 1: Table S5).

### Clinical features of OC from *FANCI* c.1813C>T carriers are similar to those of HGSC cases

We reviewed available clinical characteristics of OC in *FANCI* c.1813C>T carriers. Given the paucity of *FANCI* variants, we focused on 13 FC OC carriers of *FANCI* c.1813C>T from familial and sporadic FC OC study groups. The seven carriers found in the context of sporadic OC cases (Additional file 1: Table S12) were reported as HGSC subtype. The remaining six carriers were identified in OC cases with a known family history of cancer (Additional file 1: Table S12), where five had serous subtype OC and one had a mucinous subtype OC. There appeared to be no striking differences in the ages of the diagnosis for OC in carriers where age ranged from 40 to 81 years (average = 59.2 years) as compared with non-carriers in the sporadic OC group (average = 61 years, range 36–81 years) (Additional file 1: Table S12). Similarly, AUS HGSC *FANCI* carriers were diagnosed with OC between the ages of 31–82 years (average = 60 years) (Additional file 1: Table S12). Although sample size was limited, age range of carriers in FC cases was consistent and aligned with average age of diagnosis of OC in the North American population [1].

Available survival data showed that all seven *FANCI* c.1813C>T carriers from the sporadic OC group were deceased by the time of our investigation. They had an average survival of 61.1 months (range 9–163). Due to sample size, we were unable to perform survival analysis using Kaplan-Meier estimation as 57% (4/7) OC cases did not survive past five years (Additional file 1: Table S12). The two carriers with survival past 61 months (2/7; 28%) is comparable to survival of non-carrier sporadic HGSC cases (100/334; 30%).

### FANCI protein is expressed at low-to-moderate levels in HGSC tumour samples

**Fig. 4:**
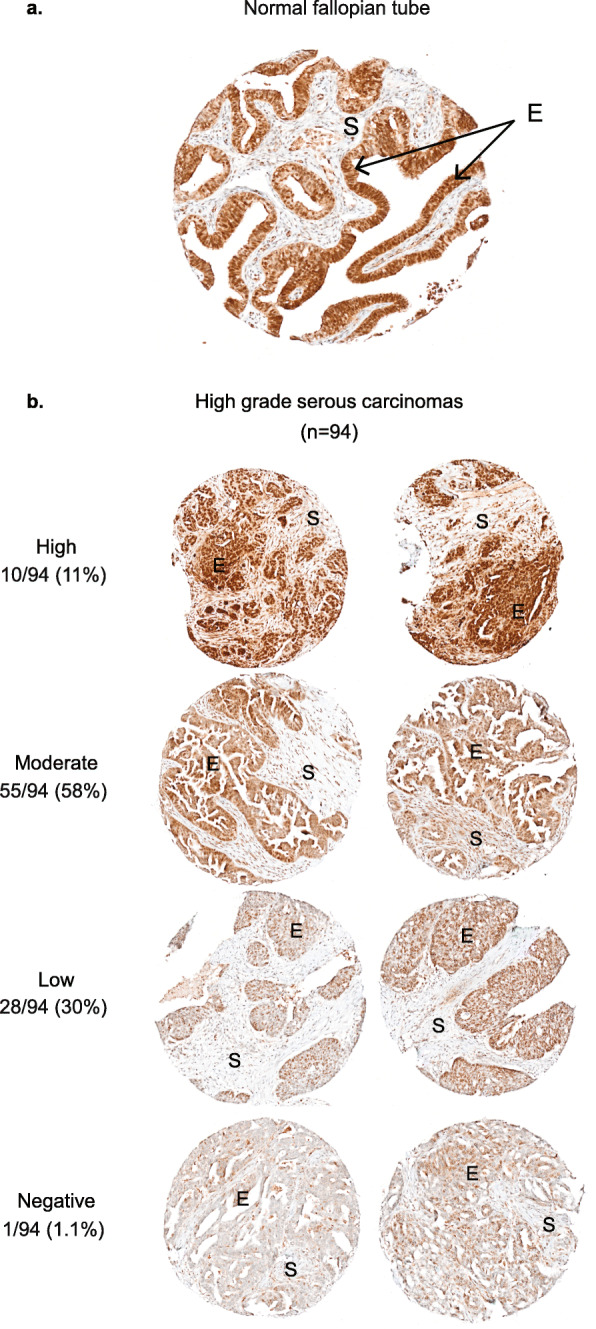
FANCI protein expression in HGSC by immunohistochemical analysis (IHC) of tissue microarrays. **a** An example of IHC analysis of FANCI protein of a paraffin-embedded normal fallopian tube tissue core. **b** Examples of different patterns of intensity of IHC analysis of FANCI protein of HGSC tissue cores in which the epithelial component is scored. E: epithelial component; S: stromal component

We performed IHC analysis of an available TMA containing cores from FFPE HGSC tumour tissues and FTE cells, a proposed tissue of origin for the HGSC subtype [100–106], staining for FANCI protein. Though a dualistic origin has been proposed for HGSC [107–109], we were only able to study FTE tissue. IHC analysis revealed strong nuclear and low-to-moderate cytoplasmic staining in FTE cells, in contrast to stromal cell components where staining was low or undetectable (Fig. 4a, Additional file 3: Fig. S4). In contrast, IHC analysis of tumour cells in HGSC tissue cores exhibited variable staining (Fig. 4b, Additional file 3: Fig. S4), where the majority (83/94, 88.3%) exhibited low-to-moderate nuclear and cytoplasmic staining in epithelial components, compared to the stromal cell components where staining intensity was low or undetectable. Using Kaplan-Meier survival analysis, we found no correlation of staining intensity in epithelial tumour cell components of the HGSC tissue cores with overall or disease-free survival (Additional file 3: Fig. S4). Age at diagnosis, disease stage, residual disease, chemotherapy type, and survival (disease-free and 5-year) were not correlated with the intensity of protein staining.

A separate IHC analysis of tumour tissues available from eight *FANCI* c.1813C>T carriers revealed a range of staining intensity (Additional file 3: Fig. S4), consistent with the expectation that the variant encoded protein could be expressed in tumours (Fig. 2a). We were not able to similarly investigate by correlative or Kaplan-Meier analyses *FANCI* variant c.1813C>T carriers due to the small number of cases.

### *FANCI* mRNA expression is associated with survival in TCGA OC cases

**Fig. 5:**
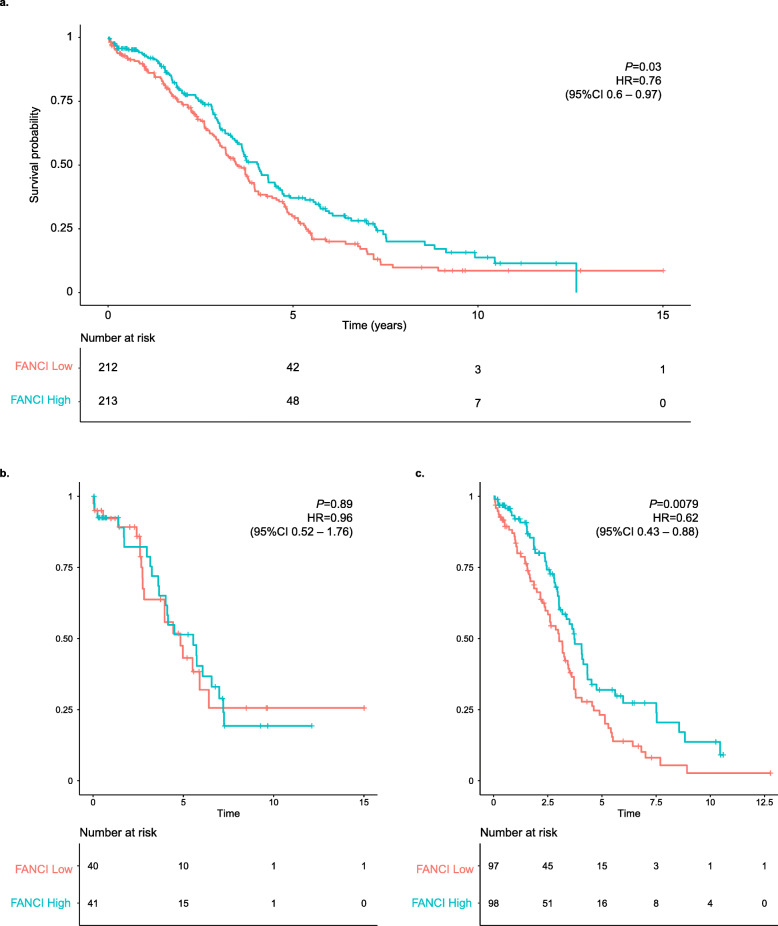
Kaplan-Meier overall survival curves of OC cases from TCGA Pan-Cancer for *FANCI* mRNA expression. All cases (**a**), *BRCA1* or *BRCA2* pathogenic variant carriers (**b**), and *BRCA1* and *BRCA2* pathogenic variant carrier negative cases (**c**) are shown. Samples were dichotomized into high and low *FANCI* expression groups based on the median

Using Kaplan-Meier analysis of TCGA Pan-Cancer cases, we found that adrenocortical cancer, kidney chromophobe, lower-grade glioma, lower-grade glioma and glioblastoma, lung adenocarcinoma, melanoma, mesothelioma, pancreatic cancer, and sarcoma along with OC were showed significant association with survival for *FANCI* mRNA expression (Additional file 1: Table S13). OC cases with high *FANCI* mRNA expression had significantly better overall survival compared to cases with low *FANCI* mRNA expression (Fig. 5a). We found that *BRCA1* and *BRCA2* pathogenic variant carriers did not show this survival benefit (Fig. 5b) and non-carriers had a stronger survival signal (Fig. 5c).

Ten rare *FANCI* variants were identified in 18/412 (4.4%) TCGA OC cases from germline WES data, where four variants are candidates based on in silico tools, including c.1813C>T (Additional file 1: Table S14). Six carriers of c.1813C>T were identified (1.5%), which is comparable to the frequency of carriers identified in the FC sporadic OC study group (1.6%). Of the 10 carriers of the 4 candidate *FANCI* variants, 3 cases had co-occurring pathogenic variants in *BRCA1* (*n* = 1) or *BRCA2* (*n* = 2). No *FANCI* carriers had co-occurring candidate variants in *BRIP1*, *RAD51C*, or *RAD51D*. Age of diagnosis was similar to FC OC cases ranging from 38 to 81 (average = 58.9; *n* = 9 cases).

## Discussion

*FANCI* c.1813C>T was the only candidate *FANCI* variant identified in our study of FC OC cases. Our strategy for the discovery of new CPGs in OC was predicated upon the genetic drift observed in FCs of Quebec and thus the expectation that candidate risk alleles frequently occur and can be readily be identified due to common ancestors in this population [25, 26]. Our findings are reminiscent of the identification of specific variants in familial FC cancer populations of Quebec, such as *RAD51D* c.620C>T; p.S207L in familial and sporadic OC cases [33], *PALB2* c.2323C>T; p.Q775X in BC cases and HBC families [32, 110], and *MSH6* c.10C>T; p.Q4X in colorectal cancer (Lynch Syndrome) families [111]. Given the unique genetic architecture of the FC population of Quebec, it is likely that carriers of *FANCI* c.1813C>T have common ancestors as has been shown with carriers of frequently occurring pathogenic variants in *BRCA1* [40, 112], *BRCA2* [40, 92, 113], and *MSH6* [111] in cancer families. As expected, given the genetic heterogeneity observed with the above examples of CPGs in non-FC populations, we identified 10 candidate *FANCI* variants in AUS HGSC cases and 4 in TCGA OC cases, which included our *FANCI* variant. Although a recent genome-wide discovery study of AUS HGSC cases did not report *FANCI* among the list of potential new CPGs for OC [47], missense variants were not investigated [47].

*FANCI* c.1813C>T might exert its deleterious effect as a hypomorphic variant, as suggested by the instability of the encoded isoform in our cell line models, which include OC cell lines. Though tumour DNA was not available for all of our variant carriers, Sanger sequencing of DNA from FFPE tumour cells suggest loss of the WT allele and retention of the variant allele had occurred in two FC HGSC *FANCI* c.1813C>T carriers, as shown in Additional file 3: Fig. S5. Interestingly, tumour samples from a bilateral OC case predominantly exhibited the *FANCI* variant allele suggesting that loss of the WT allele could have been be an early event in tumour progression in this case. Also, HGSC samples from both cases had acquired somatic pathogenic variants in *TP53*, a known major driver of tumourigenicity in the majority of HGSCs [114, 115]. Our IHC analyses showed differential FANCI protein expression, with a high proportion of HGSC tumour cells exhibiting low-to-moderate levels of protein expression. This is in contrast to consistent FANCI protein expression observed in FTE cells. These findings suggest loss of *FANCI* may play a role in OC akin to that suggested by other CPGs in the HR pathway, such as *BRCA1* and *BRCA2* [116]. In light of the dualistic origin of epithelial OC [107–109], future studies involving ovarian surface epithelial cells could also define the role of FANCI in OC. Results from analyses of TCGA data also suggest the role of *FANCI* in OC where OC cases with higher *FANCI* mRNA expression had a better overall survival outcome. In keeping with this hypothesis is that loss of the chromosome 15q arm, which contains the *FANCI* locus (15q26.1), has been reported in 55% of 978 HGSC samples by TCGA project [115]. Though the curves of the TCGA Kaplan-Meier plots are separated at the 5-year mark, future analyses of a large sample group, focusing on 5-year survival, could potentially have more clinical relevance as the majority of HGSC patients (> 75%) are deceased in this time period.

The highest frequency of carriers was in *BRCA1* and *BRCA2* pathogenic variant negative OC index cases from OC families (13%), when also including the multi-case discovery family in this group, which is significantly higher compared to sporadic OC cases (*P* = 0.01, Fisher’s exact). Variant carriers in *BRCA1* and *BRCA2* pathogenic variant negative OC families were also more frequent when compared to cancer-free FC females by including the OC discovery family in our analysis (*P* = 0.02, Fisher’s exact).

Based on available genetic data from non-Finnish EURs, the allele frequency of *FANCI* c.1813C>T at 1% is higher than expected as compared to many pathogenic variants in established CPGs. Similarly, the carrier frequency of c.1813C>T in AUS cancer-free controls at 1.9% was more common than anticipated. The carrier frequency of c.1813C>T in the general population is reminiscent of the pathogenic *CHEK2* c.1100delC, a moderate-risk BC-predisposing variant, which also has a similarly high carrier frequency of 1.4% in population controls as compared with other pathogenic variants in known CPGs for BC and OC [117]. This *CHEK2* variant was also found more frequently in BC cases from HBC families than sporadic BC cases, relative to healthy controls [117]. Although our estimates of overall risk to OC using OCAC data was inconclusive, carriers of *FANCI* c.1813C>T in FC *BRCA1* and *BRCA2* pathogenic variant negative OC families have an increased risk based on the OR of 5.8 (95%CI = 1.7–20; *P* = 0.005). Though the confidence interval is wide, due to the small sample size, our findings are supported by the observation that cancer-free *FANCI* c.1813C>T carriers (female/male) were more likely to have a first-degree relative with OC in the FC population.

Given the allele frequencies observed among OC cases and controls, it is clear that penetrance is low for *FANCI* variant carriers. Although we cannot obtain a precise estimate given the numbers of carriers available, penetrance for *FANCI* will evidently be much lower than penetrance for pathogenic variants in *BRCA1* and *BRCA2*. Assuming that *FANCI* is a risk variant for OC, it is possible that other variants modify this risk. Although we did not identify other strong candidates in our WES analyses, it may be possible in the future to estimate a polygenic risk for OC based on a set of common variants, and then to explore the *FANCI*-associated risk of OC after controlling for the polygenic background, as has been done for BC and other diseases [118]. Similarly, the effect of risk modifiers in the CARTaGENE cancer-free controls in the context of *FANCI* variant carriers is unknown. We are mindful of the fact that FC cancer cases were recruited during a different time period than FC cancer-free controls, and it is possible that risk modifiers could be different across these groups, though this information is not available for FC cancer cases.

It is interesting that *rs8037137*, which is located 1.68 mega-base pairs downstream of *FANCI* c.1813C>T, was among the polymorphic genetic markers found significantly associated with risk to either invasive epithelial or HGSC subtype OC in a large genome-wide association analysis of OCAC data [44]. Consistent with these findings is our observation that the OR for *FANCI* c.1813C>T and c.824T>C in the OCAC study groups are highest in endometrioid and HGSC subtype OC cases. A similar analysis of other candidate *FANCI* variants identified in our study was not possible as corresponding genetic data was not available in the OCAC database. The possibility that *FANCI* c.1813C>T is a moderate-risk allele with variable penetrance is consistent with our observations, though we are mindful of the limitations of our study due to sample size. Based on the allele frequency, we would require an estimated sample size of approximately 100 OC families and 7000 female cancer-free controls or 13,000 HGSC cases and 115,000 female cancer-free controls to achieve 80% power, numbers that are currently unattainable in FCs.

During the course of this investigation, *FANCI* loss-of-function and missense variants in a targeted analysis of selected DNA repair genes in OCAC cases (*n* = 6385) and controls (*n* = 6115) were reported, where only *PALB2* showed significant differences [119]. Based on sample size, the study was not sufficiently powered to identify moderate-risk alleles. Interestingly, 49 candidate *FANCI* variants, including loss-of-function variants (frameshift, nonsense, and splicing), and missense variants were reported (see Additional file 1: Table S15 and Additional file 3: Fig. S6). Although we were able to analyse *FANCI* c.1813C>T in OCAC, this variant was not listed among the candidates, as only variants with VAF < 1% were investigated in this study.

Although *FANCI* c.1813C>T variant carriers were found in FC familial BC cases, there were proportionally more carriers in BC cases from HBOC families than in HBC families. We also identified a variant carrier in a BC family of Greek Canadian origin, a family from the same catchment area as our FC cancer families. These findings are in part reminiscent of the variable penetrance for BC and OC for known high-risk CPGs, where carriers are more likely to harbour pathogenic variants in *BRCA1* or *BRCA2* (or *PALB2*) based on family history of BC and OC [40]. There have been independent reports of BC cases carrying other *FANCI* variants with VAF 10^−3^ to 10^−6^ in cancer-free individuals. At least 19 different variants have been described in familial and/or sporadic BC cases: four nonsense, three frameshift, two splicing, and 10 missense (see Additional file 1: Table S16 and Additional file 3: Fig. S7) [120–127]. These *FANCI* variants were reported in Finnish [125] (4/1524, 0.3%), Chinese [124] (1/99, 1%), and two Spanish [122, 126] (1/154, 0.6% and 1/94, 1.1%) studies. The role of *FANCI* in other cancer types remains to be determined, though there have been reports of *FANCI* variant carriers in a variety of cancer types such as prostate cancer [125, 128, 129], sarcoma [130], malignant pleural mesothelioma [131], acute myeloid leukaemia [132], head and neck carcinoma [133], and colorectal cancer [134] (see Additional file 1: Table S17 and Additional file 3: Fig. S7).

FANCI regulates the recruitment of the FA core complex to sites of interstrand crosslinks, and thus plays an important function upstream in the FA-HR DNA repair pathway [135]. FANCI encodes one of only two proteins that comprise the ID2 complex, the other being FANCD2. In cellulo modeling using cell lines, pathogenic variants or gene knockouts of *BRCA1*, *PALB2*, or *RAD51D* have exhibited sensitivity to cisplatin and PARPi's, providing some insight into their role in DNA repair [33, 136–138]. We observed sensitivity to cisplatin but not to the PARPi olaparib in cell lines expressing the FANCI p.L605F isoform. Although the mechanism is unknown, these findings are consistent with a report showing lack of sensitivity to a PARPi (KU0058948) in a fibroblast cell line transduced with HPV E6/E7 from a *FANCI* FA patient, as well as in cell lines generated from *FANCA*, *FANCL*, *FANCD2*, and *FANCJ* (*BRIP1*) patients [84]. The indirect role of FANCI in HR DNA repair and recent evidence suggesting that FANCI also has functions independent from the FA DNA repair pathway [139–143] may be consistent with our in cellulo studies. Further investigation of FANCI p.L605F in additional cell lines, including normal cell lines which are more karyotypically normal such as those that are representative of the various origins of epithelial OCs, could lend support to the influence of this variant on protein function in this cancer context.

Biallelic inactivation of *FANCI* has been associated with FA, a rare autosomal recessive disease that is characterized by congenital defects and developmental disabilities [36–38]. FA is a heterogenous genetic disease with 22 known causal genes, where *FANCI* implicated cases comprise approximately 1% of all FA diagnoses [144]. No clear genotype/phenotype association has been identified for *FANCI*-associated FA, though 7/16 (44%) patients show at least three features of the VACTERL-H association [145], which is a disease characterized by a non-random association of birth defects (typically at least three) affecting multiple parts of the body. *FANCI* c.1813C>T; p.L605F has been reported in ClinVar as benign or likely benign (*n* = 6 submissions) in the context of *FANCA* associated FA (*n* = 1), *FANCI*-associated FA (*n* = 2), or unspecified conditions (*n* = 3) with only in silico (no in cellulo or in vitro) evidence provided to classify this variant and no information on zygosity in carriers nor cancer context. Mild or no FA phenotypes have been observed for other homozygous hypomorphic variants in FA genes (*BRCA1* (*FANCS*), *BRCA2* (*FANCD1), FANCA*, and *PALB2* (*FANCN*)) [146–149]. Hypomorphic variants in *RB1*, the causative gene of retinoblastoma have been found to confer significantly lower penetrance (< 25%) as compared to more common loss-of-function variants which are highly penetrant (> 95%) for the disease [150]. As *FANCI*-associated FA cases are rare, the incidence of cancer in biallelic carriers has not been reported. Heterozygous carriers of *FANCI* c.1264G>A; p.G422R, a pathogenic variant that has been reported in two *FANCI*-associated FA cases [38, 151], were identified in AUS cases and controls in our study. Although there was no information about cancer incidence, a Fanci KO mouse model was recently reported describing phenotypes consistent with developmental defects, though they also reported a low Mendelian ratio [152].

## Conclusions

This is the first study to describe candidate variants in *FANCI* in the context of familial OC and in a member of the ID2 complex of the FA DNA repair pathway. Our strategy of investigating a limited number of familial and sporadic cancer cases from a population showing genetic drift found an increased frequency of carriers in OC cases. In cellulo and in vitro analysis of a missense variant found to recur in cancer cases implicates *FANCI* as a new candidate OC-predisposing gene. This study emphasizes the importance of pursuing missense variants during the gene discovery phase, especially when plausible candidates are revealed by analyses of defined cancer families. Indeed, a large number of pathogenic variants in known CPGs, such as *BRCA1* and *BRCA2*, are missense variants where they have been vetted using in cellulo and/or in vitro functional studies [31]. Although some of the identified *FANCI* variants are predicted to affect gene function as shown by in cellulo analyses of FANCI p.L605F isoform, further studies are warranted to evaluate their role in OC risk. Our study suggests the possibility that *FANCI* variants might confer moderate risk to OC akin to *CHEK2* variants to BC risk and question the classification of *FANCI* c.1813C>T as benign or likely benign but support that it is likely pathogenic [77]. We were not able to estimate penetrance due to sample size and inability to perform extensive familial studies associating carrier status with affected cases as has recently been performed with *PALB2* risk [153]. Establishing risk is important in the context of familial aggregations of OC and host behaviours known to affect risk, such as has been shown with oral contraceptive pill use in carriers of pathogenic *BRCA1* and *BRCA2* variants. Risk-reducing surgery may not be necessary for *FANCI* variant carriers having significantly reduced risk due to oral contraceptive pill usage [154]. An investigation of carriers of candidate *FANCI* variants is also warranted given the intriguing observation of sensitivity to cisplatin but not to olaparib in the in cellulo studies of *FANCI* c.1813C>T, as this might impact the efficacy of PARPi’s in the treatment of HGSC in these cases.

## Supplementary Information

**Additional file 1.** All supplementary tables referenced in the manuscript. Tables S1-S17.

**Additional file 2 **Supplementary note describing the genetic analyses of *POLG* c.2492A>G found to be in linkage with *FANCI* c.1813C>T carriers.

**Additional file 3.** All supplementary figures referenced in the manuscript. Figures S1-S7.

**Additional file 4.** All full blots associated with Figure 2, Fig, S1, and Fig. S2.

## Data Availability

WES data for familial cases and CARTaGENE data will be returned to their respective biobanks at the conclusion of our study of *FANCI* which is still ongoing. For more information concerning these data, contact Patricia N. Tonin at patricia.tonin@mcgill.ca. WES data for AUS cases is available as stated in the original publication. For CDN data contact Marc Tischkowitz at mdt33@cam.ac.uk. The data sets used for TCGA, OCAC, BCAC, and gnomAD analyses are available from each data resource bank.

## Abbreviations

OC: Ovarian cancer
HGSC: High-grade serous carcinoma
PARPi: Poly (ADP-ribose) polymerase inhibitors
CPG: Cancer-predisposing gene
FA: Fanconi anaemia
CI: Confidence interval
BC: Breast cancer
EUR: Europe
FC: French Canadian
WES: Whole exome sequencing
HR: Homologous recombination
ICL: Interstrand crosslink
CDN: Canadian non-FC
AUS: Australian
PBL: Peripheral blood lymphocyte
FFPE: Formalin-fixed paraffin-embedded
TMA: Tissue microarray
RRCancer: Réseau de recherche sur le cancer
MUHC: McGill University Health Centre
BWA: Burroughs–Wheeler algorithm
dbSNP: Single Nucleotide Polymorphism database designation
SIFT: Sorting Intolerant from Tolerant
NHLBI: National Heart, Lung and Blood Institute
ESP: Exome Sequencing Project
MGC: McGill Genome Centre
IGV: Integrative Genomics Viewer
VAF: Variant allele frequency
gnomAD: Genome Aggregation Database
HBC: Hereditary breast cancer
HBOC: Hereditary breast and ovarian cancer
PBWT: Positional Burroughs–Wheeler transformation
GERP++: Genomic Evolutionary Rate Profiling
PhyloP: Phylogenetic *P* values
PhastCons: Phylogenetic Analysis with Space/Time models Conservation
SiPhy: Site-specific Phylogenetic analysis
CADD: Combined Annotation Dependent Depletion
Condel: Consensus Deleteriousness
MetaLR: Meta-analytic Logistic Regression
MetaSVM: Meta-analytic Support Vector Machine
VEST: Variant Effect Scoring Test
REVEL: Rare Exome Variant Ensemble Learner
MaxEntScan: Maximum Entropy Modeling of Short Sequence Motifs
dbscSNV: Database Splicing Consensus Single Nucleotide Variant
ADA: AdaBoost
RF: Random Forest
OCAC: Ovarian Cancer Association Consortium
BCAC: Breast Cancer Association Consortium
SE: Standard error
TCGA: The Cancer Genome Atlas
KO: Knockout
MMC: Mitomycin C
CHX: Cycloheximide
WT: Wild type
EV: Empty vector
SEM: Standard error of the mean
IHC: Immunohistochemistry
FTE: Fallopian tube epithelial
SPSS: Statistical Package for the Social Sciences
KD: Knockdown
