# Supplementary material for: A functionally impaired missense variant identified in French Canadian families implicates FANCI as a candidate ovarian cancer-predisposing gene
Source: Genome Med. 2021 Dec 3;13:186. doi: 10.1186/s13073-021-00998-5 (PMC8642877; doi:10.1186/s13073-021-00998-5)
Supplement: Supplementary file 2 — Additional file 2 Supplementary note describing the genetic analyses of POLG c.2492A>G found to be in linkage with FANCI c.1813C>T carriers. [file 13073_2021_998_MOESM2_ESM.pdf]

Supplementary Note

To further characterize the germline genomic landscape of *FANCI* c.1813C>T carriers, we performed WES analysis of all seven carriers identified in the sporadic FC OC cases and compared it with WES data from five carriers identified in OC families (**Table 1**). We investigated DNA repair pathways genes (n=276 (1)) rationalizing that aberrant DNA repair is a hallmark of cancer and other variants in these genes may contribute to risk if shared among *FANCI* c.1813C>T carriers. Bioinformatic analyses of WES data identified a rare DNA polymerase γ (*POLG*) c.2492A>G; p.Y831C variant in the heterozygous state in all *FANCI* c.1813C>T carriers. *POLG* encodes the catalytic subunit for polymerase γ, the only known mitochondrial DNA polymerase, and has not been reported in association with cancer (2). *POLG* c.2492A>G has not been reported in association with a disease. Interestingly, *POLG* c.2492A>G is predicted to be damaging in 10/13 *in silico* tools (data not shown). Unlike *FANCI*, *POLG* is transcribed on the negative strand immediately downstream of *FANCI* where its 3' UTR is encoded in part by a genetic region shared in common with *FANCI*. As the estimated distance between *FANCI* c.1813C>T and *POLG* c.2492A>G is 36.6 kilobase pairs, it is possible that these alleles are in linkage disequilibrium in the FC population due to common ancestry (3,4). To investigate this possibility, all FC cancer cases were genotyped for *POLG* c.2492A>G carrier status. Only one case was found not to carry co-occurring *FANCI-POLG* variants: a *FANCI* variant carrying mucinous OC case. We also investigated available genotyping data from CARTaGENE for cancer-free FCs and found that *FANCI* c.1813C>T tended to co-occur with *POLG* c.2492A>G (Log2 Odds Ratio=15.2) suggesting that these rare alleles are likely in linkage disequilibrium in FCs.

References

1. Knijnenburg TA, Wang L, Zimmermann MT, Chambwe N, Gao GF, Cherniak AD, et al. Genomic and molecular landscape of DNA damage repair deficiency across The Cancer Genome Atlas resource. Cell Rep. 2018;23:239–54.
2. Walker RL, Anziano P, Meltzer PS. A PAC containing the human mitochondrial DNA polymerase gamma gene (POLG) maps to chromosome 15q25. Genomics. 1997;40(2):376–8.
3. Laberge A-M, Michaud J, Richter A, Lemyre E, Lambert M, Brais B. Population history and its impact on medical genetics in Quebec. Clin Genet. 2005;68:287–301.
4. Scriver CR. Human genetics: Lessons from Quebec populations. Annu Rev Genomics Hum Genet. 2001;2:69–101.
