# Supplementary material for: A functionally impaired missense variant identified in French Canadian families implicates FANCI as a candidate ovarian cancer-predisposing gene
Source: Genome Med. 2021 Dec 3;13:186. doi: 10.1186/s13073-021-00998-5 (PMC8642877; doi:10.1186/s13073-021-00998-5)
Supplement: Supplementary file 3 — Additional file 3. All supplementary figures referenced in the manuscript. Figures S1-S7. [file 13073_2021_998_MOESM3_ESM.pdf]

Fig. S1

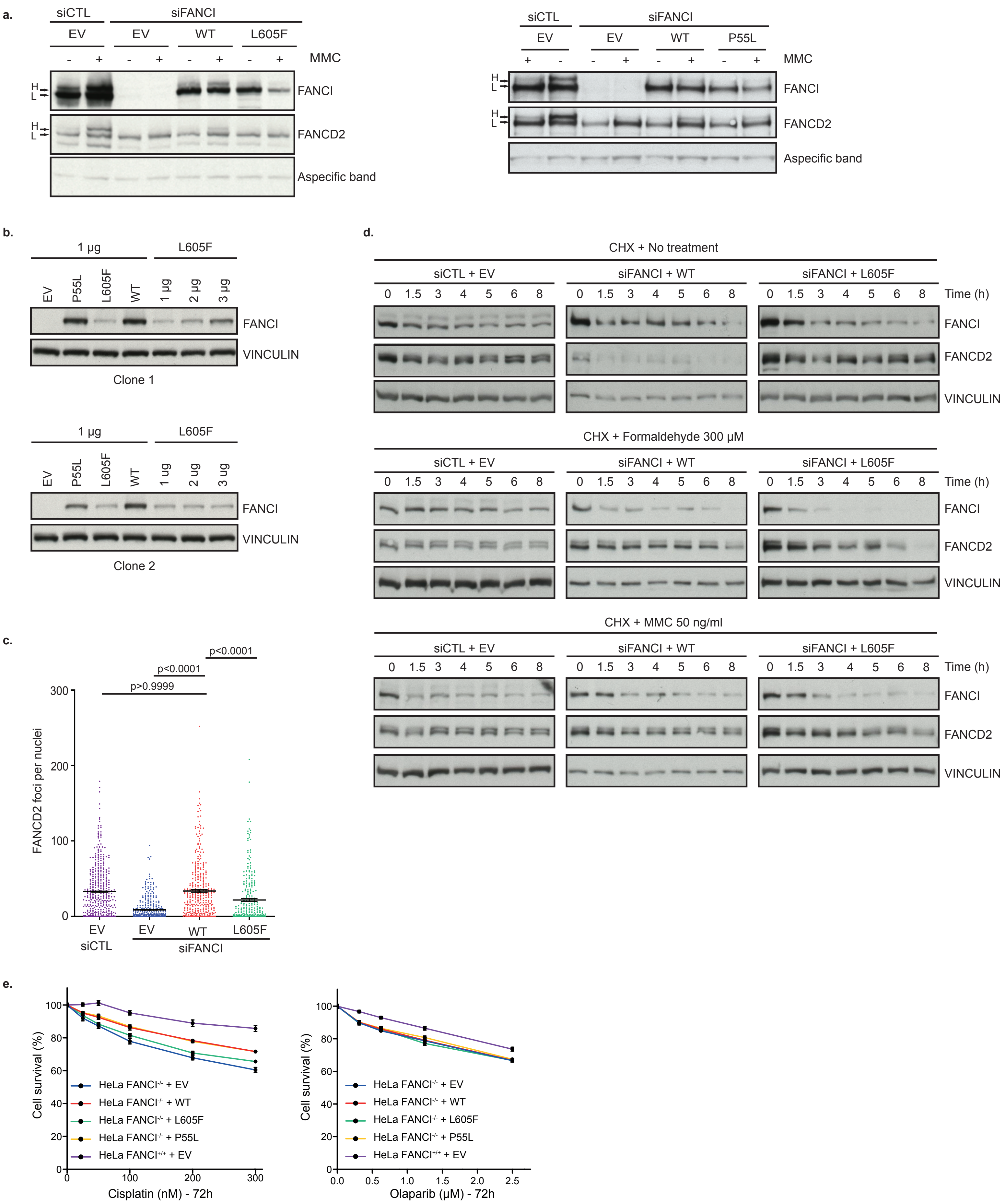
a.
siCTL
siFANCI
EV
EV
WT
L605F
MMC
H
L
FANCI
FANCD2
Aspecific band
siCTL
siFANCI
EV
EV
WT
P55L
MMC
FANCI
FANCD2
Aspecific band
b.
1 µg
L605F
EV
P55L
L605F
WT
1 µg
2 µg
3 µg
FANCI
VINCULIN
Clone 1
Clone 2
c.
p<0.0001
p<0.0001
p>0.9999
FANCD2 foci per nuclei
300
200
100
0
EV
EV
WT
L605F
siCTL
siFANCI
d.
CHX + No treatment
CHX + Formaldehyde 300 µM
CHX + MMC 50 ng/ml
siCTL + EV
siFANCI + WT
siFANCI + L605F
0
1.5
3
4
5
6
8
Time (h)
FANCI
FANCD2
VINCULIN
e.
Cell survival (%)
Cisplatin (nM) - 72h
Olaparib (µM) - 72h
HeLa FANCI-/- + EV
HeLa FANCI-/- + WT
HeLa FANCI-/- + L605F
HeLa FANCI-/- + P55L
HeLa FANCI+/+ + EV

Fig. S1. The isoform with the p.L605F variant impairs FANCI stability and function. a) Western blots of HeLa FANCI$^{+/+}$ cells transfected with siRNA control (siCTL) or targeting FANCI (siFANCI) and then complemented with Flag-FANCI constructs or empty vector (EV) and treated with 50 ng/ml MMC for 18 hours. The upper band, H, shows the ubiquitination of FANCI and FANCD2 after treatment. The lower band, L, corresponds to non-ubiquitinated FANCI or FANCD2. VINCULIN was used as a control. b) Western blot of HeLa FANCI$^{-/-}$ cells from clones 1 and 2 were complemented with increasing amounts of FANCI p.L605F plasmid. c) Immunofluorescence of HeLa FANCI$^{+/+}$ cells transfected with siRNA targeting FANCI and complemented with EV or Flag-FANCI siRNA resistant constructs. The number of FANCD2 foci in Flag positive cells after treatment with MMC (50 ng/ml, 18 hours) is shown. The upper and lower edge of the solid bars represents the SEM. The Kruskal-Wallis test was used to compare groups and the *P*-value is shown for each test. d) Western blots of HeLa FANCI$^{+/+}$ cells transfected with siRNA targeting FANCI and complemented with Flag-FANCI siRNA resistant constructs and treated with CHX and either mock-treated or treated with damaging agents formaldehyde or MMC for different lengths of time. At each time point, whole cell extracts were analyzed by Western blot to assess protein levels. Experiment has been performed twice. e) Survival curves of HeLa FANCI$^{-/-}$ cells from clone 2 that were complemented with constructs of Flag-FANCI variants or empty vector (EV) and plated in triplicate in a 96 well plate. Cell viability was monitored following cisplatin or olaparib treatments for 72 hours and was assessed by counting remaining nuclei. Experiments were performed in three biological replicates.

Fig. S2

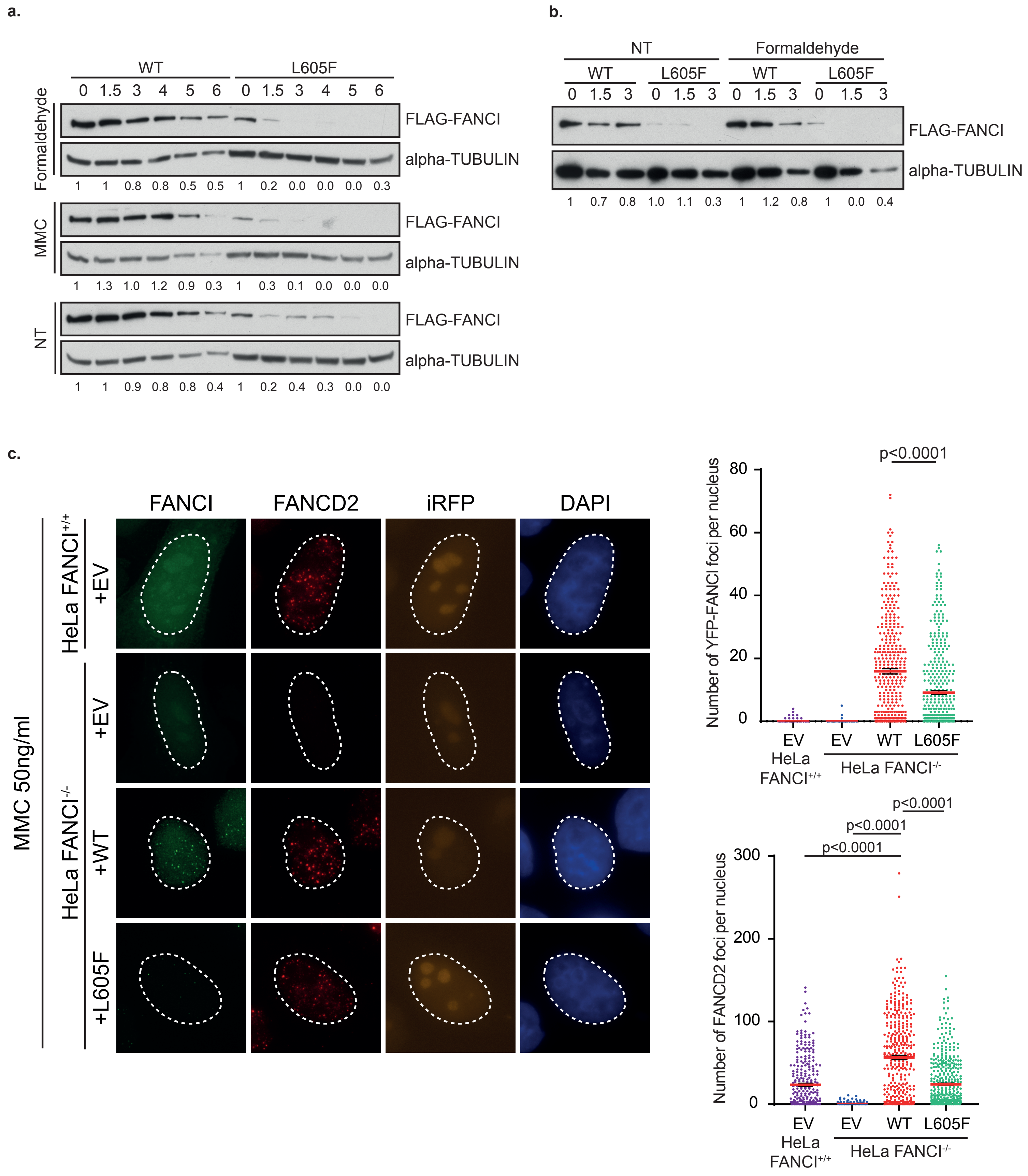

a.
WT
L605F
0 1.5 3 4 5 6 0 1.5 3 4 5 6
Formaldehyde
FLAG-FANCI
alpha-TUBULIN
1 1 0.8 0.8 0.5 0.5 1 0.2 0.0 0.0 0.0 0.3
MMC
FLAG-FANCI
alpha-TUBULIN
1 1.3 1.0 1.2 0.9 0.3 1 0.3 0.1 0.0 0.0 0.0
NT
FLAG-FANCI
alpha-TUBULIN
1 1 0.9 0.8 0.8 0.4 1 0.2 0.4 0.3 0.0 0.0
b.
NT
Formaldehyde
WT
L605F
WT
L605F
0 1.5 3 0 1.5 3 0 1.5 3 0 1.5 3
FLAG-FANCI
alpha-TUBULIN
1 0.7 0.8 1.0 1.1 0.3 1 1.2 0.8 1 0.0 0.4
c.
FANCI
FANCD2
iRFP
DAPI
MMC 50ng/ml
HeLa FANCI+/+
+EV
HeLa FANCI-/-
+EV
+WT
+L605F
Number of YFP-FANCI foci per nucleus
80
60
40
20
0
p<0.0001
EV
HeLa
FANCI+/+
EV
WT
L605F
HeLa FANCI-/-
Number of FANCD2 foci per nucleus
300
200
100
0
p<0.0001
p<0.0001
p<0.0001
EV
HeLa
FANCI+/+
EV
WT
L605F
HeLa FANCI-/-

Fig. S2. The isoform with the p.L605F variant impairs FANCI stability in OC cell lines and FANCI localization to DNA damage in HeLa cells. a) Western blots of OVCAR-4 cells expressing Flag-FANCI wild type (WT) or p.L605F. Cells were treated with CHX and either mock-treated (NT) or treated with formaldehyde (300 μM for the indicated times) or MMC (50 ng/ml for the indicated times). Alpha-tubulin was used as a loading control. b) Western blots of OVCAR-3 cells expressing Flag-FANCI wild type (WT) or p.L605F, treated with cycloheximide and either mock-treated or treated with formaldehyde (300 μM for the indicated times). Alpha-tubulin was used as a loading control. c) Immunofluorescence analysis of wild-type HeLa or HeLa FANCI$^{-/-}$ cells complemented with either YFP alone (EV), YFP-FANCI, or YFP-FANCI p.L605F constructs. iRFP was used as a transfection marker. The adjacent scatter plot shows the number of YFP-FANCI foci per nucleus or FANCD2 foci in iRFP-positive cells after treatment with MMC (50 ng/ml, 18 hours). Mean with SEM is represented. The Kruskal-Wallis test was used to compare groups and the P-value is shown for each test.

Fig. S3

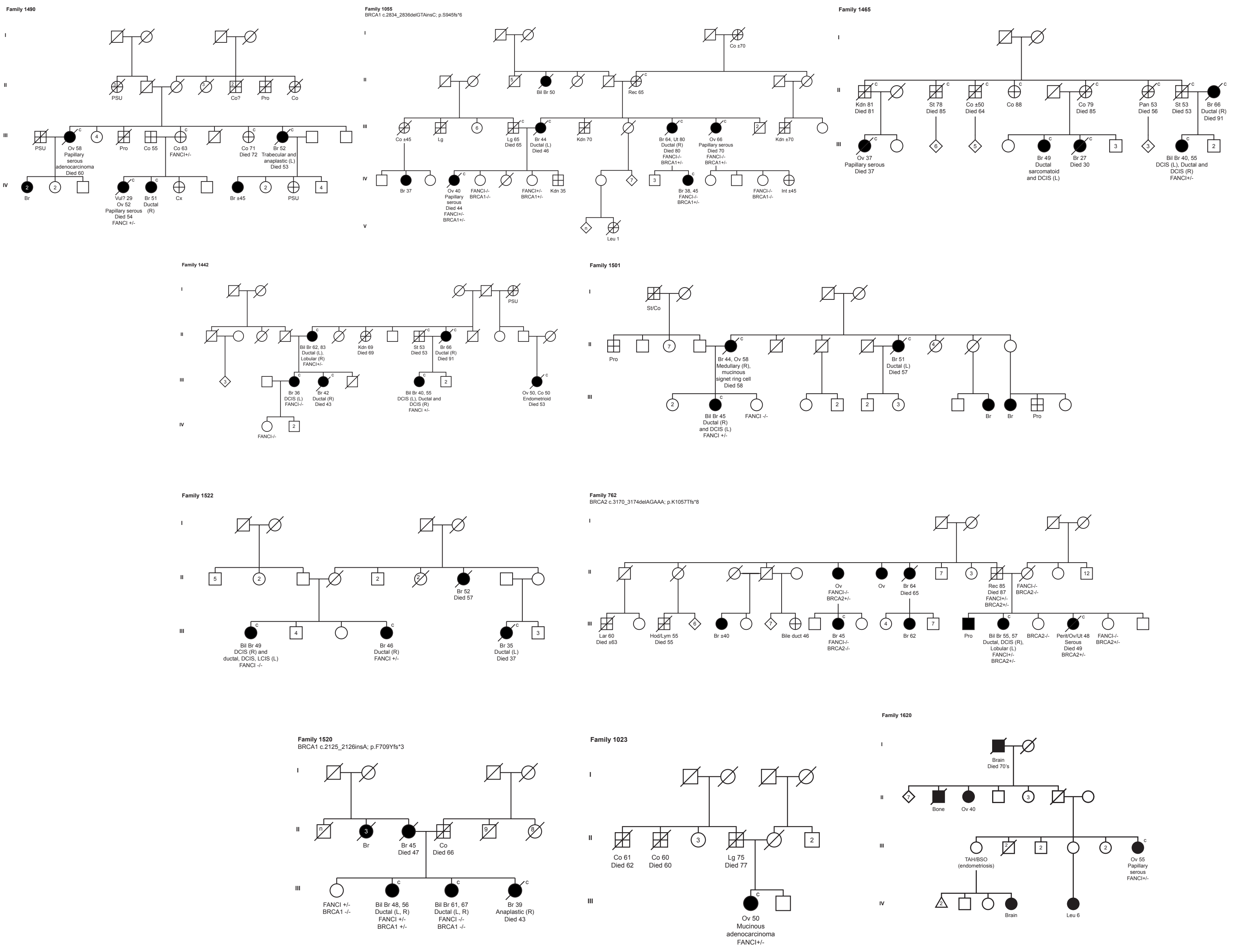

Family 1490
PSU
Co?
Pro
Co
PSU
Ov 58 Papillary serous adenocarcinoma Died 60
Pro
Co 55
Co 63 FANCI+/-
Co 71 Died 72
Br 52 Trabecular and anaplastic (L) Died 53
Br
Vul? 29 Ov 52 Papillary serous Died 54 FANCI +/-
Br 51 Ductal (R)
Cx
Br ±45
PSU
Family 1055
BRCA1 c.2834_2836delGTAinsC; p.S945fs*6
Co ±70
Bil Br 50
Rec 65
Co ±45
Lg
Lg 65 Died 65
Br 44 Ductal (L) Died 46
Kdn 70
Br 64, Ut 80 Ductal (R) Died 80 FANCI-/- BRCA1+/-
Ov 66 Papillary serous Died 70 FANCI-/- BRCA1+/-
Kdn ±70
Br 37
Ov 40 Papillary serous Died 44 FANCI+/- BRCA1+/-
FANCI-/- BRCA1-/-
FANCI+/- BRCA1+/-
Kdn 35
Br 38, 45 FANCI-/- BRCA1+/-
FANCI-/- BRCA1-/-
Int ±45
Leu 1
Family 1465
Kdn 81 Died 81
St 78 Died 85
Co ±50 Died 64
Co 88
Co 79 Died 85
Pan 53 Died 56
St 53 Died 53
Br 66 Ductal (R) Died 91
Ov 37 Papillary serous Died 37
Br 49 Ductal sarcomatoid and DCIS (L)
Br 27 Died 30
Bil Br 40, 55 DCIS (L), Ductal and DCIS (R) FANCI+/-
Family 1442
PSU
Bil Br 62, 83 Ductal (L), Lobular (R) FANCI+/-
Kdn 69 Died 69
St 53 Died 53
Br 66 Ductal (R) Died 91
Br 36 DCIS (L) FANCI-/-
Br 42 Ductal (R) Died 43
Bil Br 40, 55 DCIS (L), Ductal and DCIS (R) FANCI +/-
Ov 50, Co 50 Endometrioid Died 53
FANCI-/-
Family 1501
St/Co
Pro
Br 44, Ov 58 Medullary (R), mucinous signet ring cell Died 58
Br 51 Ductal (L) Died 57
Bil Br 45 Ductal (R) and DCIS (L) FANCI +/-
FANCI -/-
Br
Br
Pro
Family 1522
Br 52 Died 57
Bil Br 49 DCIS (R) and ductal, DCIS, LCIS (L) FANCI -/-
Br 46 Ductal (R) FANCI +/-
Br 35 Ductal (L) Died 37
Family 762
BRCA2 c.3170_3174delAGAAA; p.K1057Tfs*8
Ov FANCI-/- BRCA2+/-
Ov
Br 64 Died 65
Rec 85 Died 87 FANCI+/- BRCA2+/-
FANCI-/- BRCA2-/-
Lar 60 Died ±63
Hod/Lym 55 Died 55
Br ±40
Bile duct 46
Br 45 FANCI-/- BRCA2-/-
Br 62
Pro
Bil Br 55, 57 Ductal, DCIS (R), Lobular (L) FANCI+/- BRCA2+/-
BRCA2-/-
Perit/Ov/Ut 48 Serous Died 49 BRCA2+/-
FANCI-/- BRCA2+/-
Family 1520
BRCA1 c.2125_2126insA; p.F709Yfs*3
Br
Br 45 Died 47
Co Died 66
FANCI +/- BRCA1 -/-
Bil Br 48, 56 Ductal (L, R) FANCI +/- BRCA1 +/-
Bil Br 61, 67 Ductal (L, R) FANCI -/- BRCA1 -/-
Br 39 Anaplastic (R) Died 43
Family 1023
Co 61 Died 62
Co 60 Died 60
Lg 75 Died 77
Ov 50 Mucinous adenocarcinoma FANCI+/-
Family 1620
Brain Died 70's
Bone
Ov 40
TAH/BSO (endometriosis)
Ov 55 Papillary serous FANCI+/-
Brain
Leu 6

Fig. S3. Pedigrees of OC and BC families with *FANCI* c.1813C>T; p.L605F. Cancer type (Ov: ovarian, Leu: leukemia, Br: breast, Cx: cervical, Co: colon, Rec: rectal, St: stomach, Kdn: kidney, Pro: prostate, Lg: lung, Perit: peritoneum, Ut: uterine, Lar: larynx, Hod: Hodgkin's lymphoma, Lym: lymphoma, and PSU: primary site unknown), risk reducing surgery (TAH/BSO: total abdominal hysterectomy/bilateral salpingo-oophorectomy), and age at diagnosis is indicated. *BRCA1* and *BRCA2* carrier status is shown for pathogenic variant positive families, F762, F1055, F1520; all other families are *BRCA1* and *BRCA2* pathogenic variant negative. c next to a symbol denotes a confirmed cancer case. Pedigrees may have been truncated to protect anonymity.

Fig. S4

a.

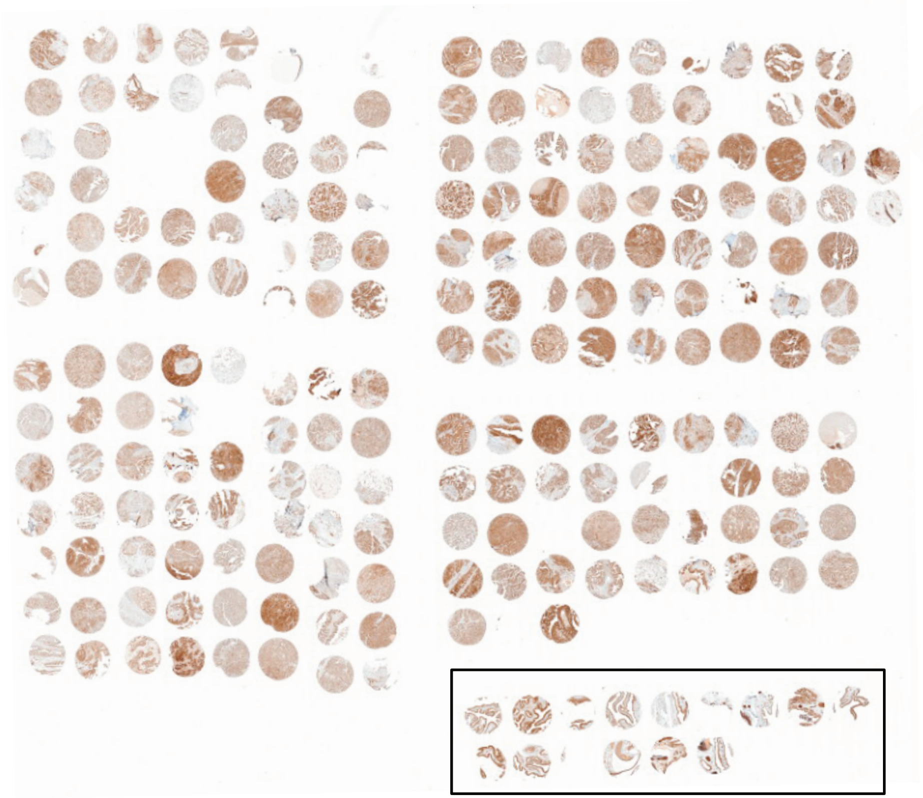

b.

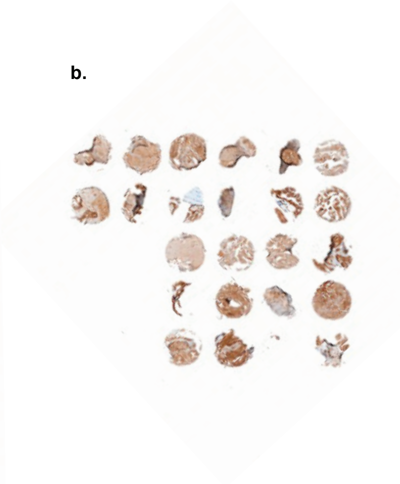

c.

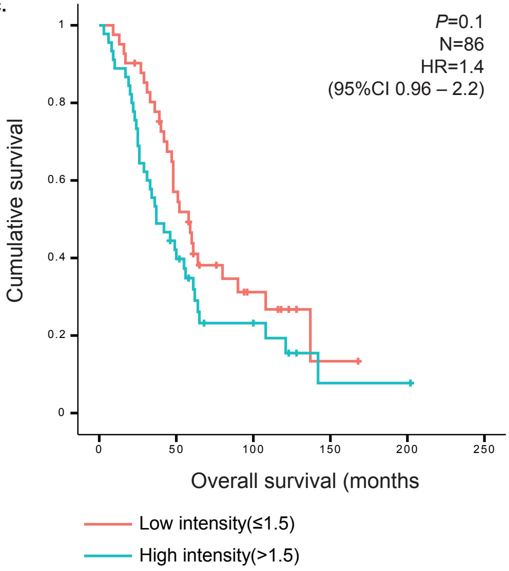
P=0.1
N=86
HR=1.4
(95%CI 0.96 – 2.2)
Cumulative survival
Overall survival (months
Low intensity(≤1.5)
High intensity(>1.5)

d.

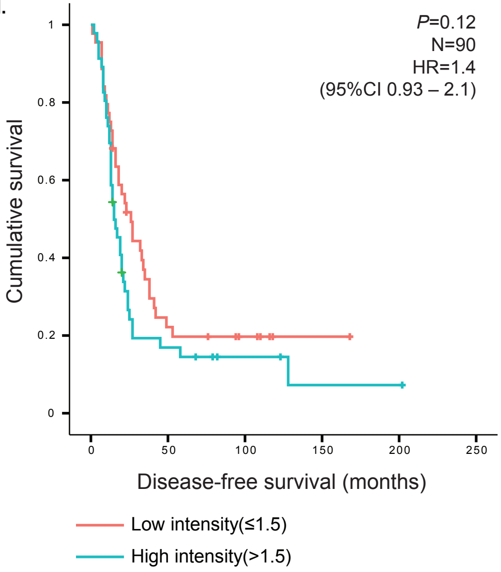
P=0.12
N=90
HR=1.4
(95%CI 0.93 – 2.1)
Cumulative survival
Disease-free survival (months)
Low intensity(≤1.5)
High intensity(>1.5)

Fig. S4. Immunohistochemical analyses of FANCI protein expression of tissue microarrays (TMAs) from (a) HGSC cases and normal fallopian tube (a, black box) and (b) *FANCI* c.1813C>T OC carriers tissue cores. TMAs were stained with anti-FANCI antibody on the same slide. Kaplan-Meier survival curve of HGSC cases for overall (c) and disease-free (d) survival (in months) as measured in the epithelial cell component. Cases included in the analyses had received only adjuvant taxol and carboplatin chemotherapy.

Fig. S5

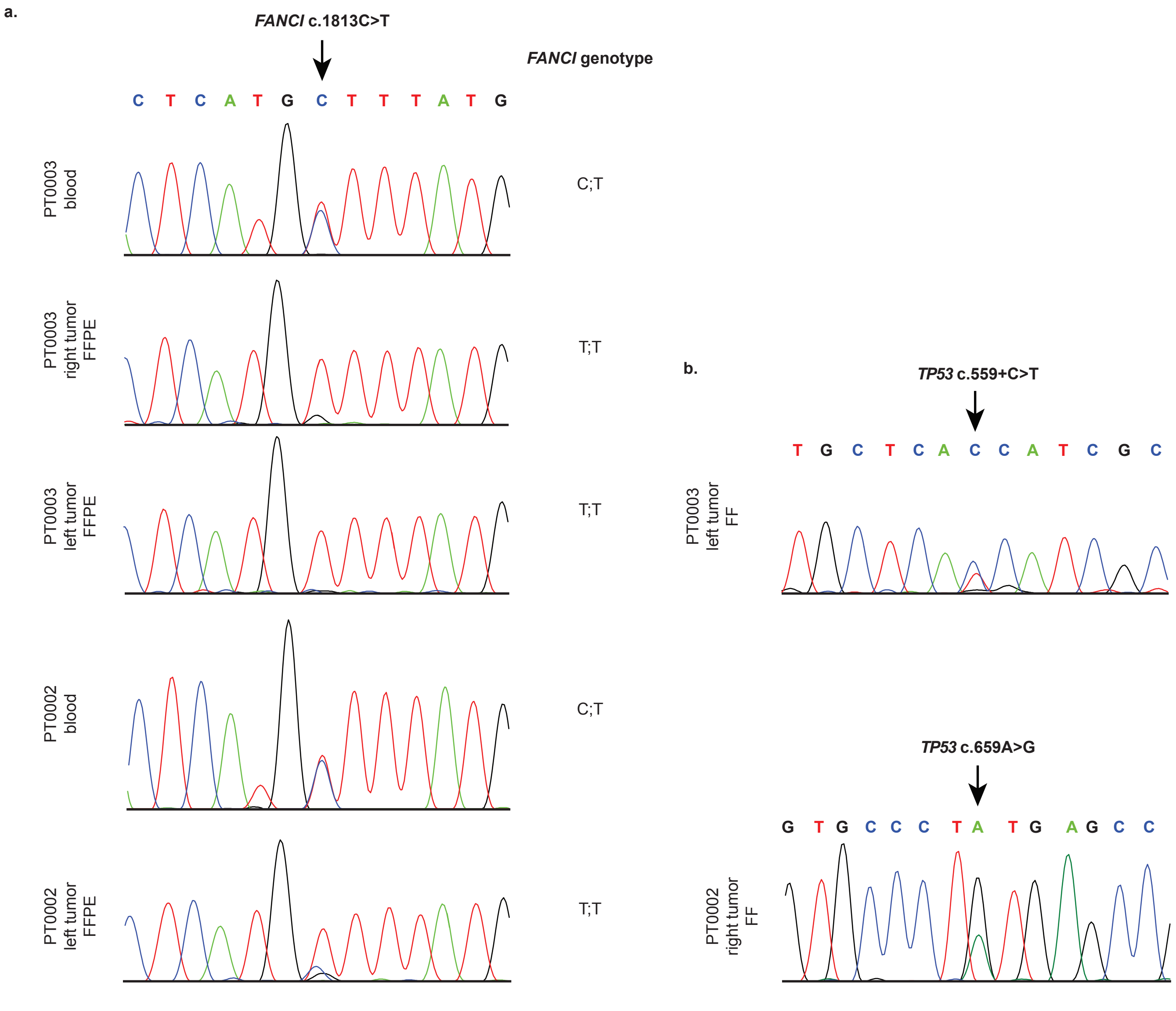
a.
FANCI c.1813C>T
FANCI genotype
C T C A T G C T T T A T G
PT0003 blood
C;T
PT0003 right tumor FFPE
T;T
PT0003 left tumor FFPE
T;T
PT0002 blood
C;T
PT0002 left tumor FFPE
T;T
b.
TP53 c.559+C>T
T G C T C A C C A T C G C
PT0003 left tumor FF
TP53 c.659A>G
G T G C C C T A T G A G C C
PT0002 right tumor FF

Fig. S5. Identification of *FANCI* c.1813C>T and *TP53* variants in French Canadian HGSC cases. a) DNA sequencing chromatogram showing the region containing c.1813C>T corresponding to DNA obtained from (top to bottom): PT0003 blood showing the heterozygous variant, PT0003 FFPE right tumor showing loss of the WT allele, PT0003 FFPE left tumor showing loss of the WT allele, PT0002 blood showing the heterozygous variant, and PT0002 FFPE left tumor showing loss of the WT allele. The forward sequence is shown. B) DNA sequencing chromatogram showing the region containing identified *TP53* variants corresponding to (top to bottom): PT0003 FF left tumor showing *TP53* variant c.559+1C>T and PT0002 FF right tumor showing TP53 variant c.659A>G. The reverse sequence is shown. FFPE: formalin-fixed paraffin-embedded tumor tissue; FF: fresh frozen tumor tissue.

Fig. S6

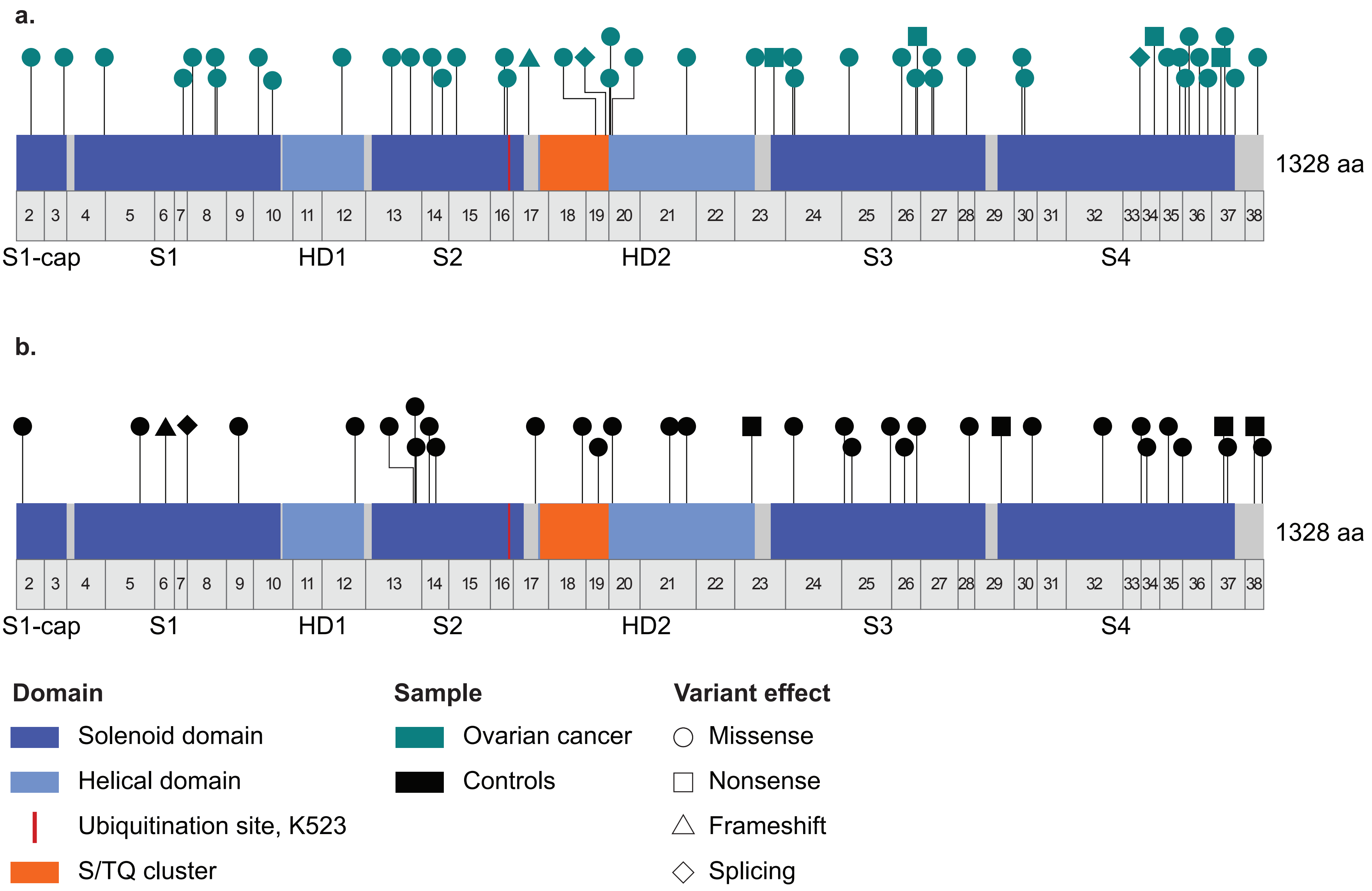
a.
1328 aa
2 3 4 5 6 7 8 9 10 11 12 13 14 15 16 17 18 19 20 21 22 23 24 25 26 27 28 29 30 31 32 33 34 35 36 37 38
S1-cap S1 HD1 S2 HD2 S3 S4
b.
1328 aa
2 3 4 5 6 7 8 9 10 11 12 13 14 15 16 17 18 19 20 21 22 23 24 25 26 27 28 29 30 31 32 33 34 35 36 37 38
S1-cap S1 HD1 S2 HD2 S3 S4
Domain
Solenoid domain
Helical domain
Ubiquitination site, K523
S/TQ cluster
Sample
Ovarian cancer
Controls
Variant effect
Missense
Nonsense
Frameshift
Splicing

Fig. S6. Schemata of *FANCI* gene showing rare variants (VAF<1%) reported in (a) OC (b) and controls from the literature. FANCI domains were adapted from pfam (https://pfam.xfam.org). *FANCI* exon locations adapted from University of California Santa Cruz Genome Browser (https://genome.ucsc.edu).

Fig. S7

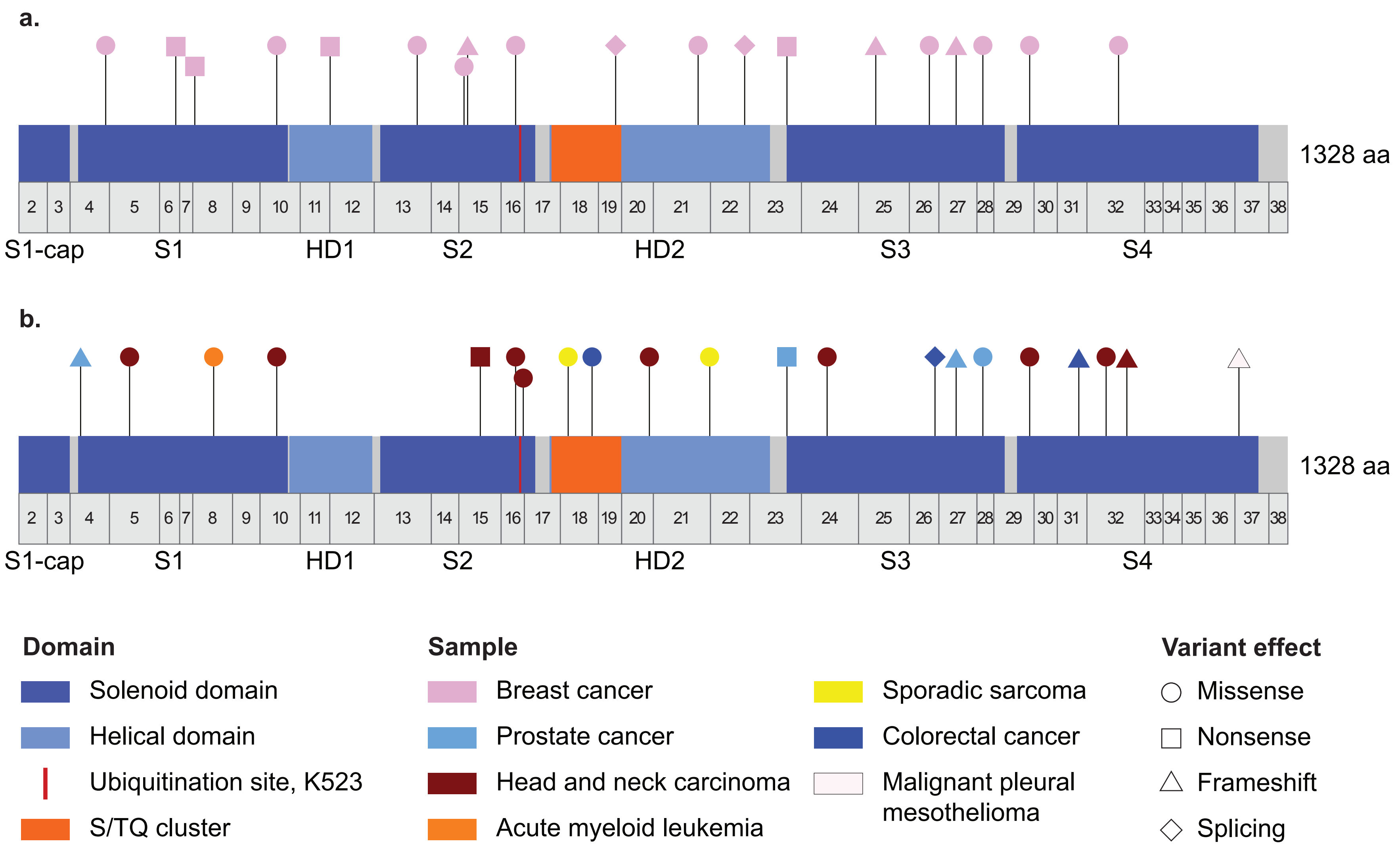
a.
1328 aa
2 3 4 5 6 7 8 9 10 11 12 13 14 15 16 17 18 19 20 21 22 23 24 25 26 27 28 29 30 31 32 33 34 35 36 37 38
S1-cap
S1
HD1
S2
HD2
S3
S4
b.
1328 aa
2 3 4 5 6 7 8 9 10 11 12 13 14 15 16 17 18 19 20 21 22 23 24 25 26 27 28 29 30 31 32 33 34 35 36 37 38
S1-cap
S1
HD1
S2
HD2
S3
S4
Domain
Solenoid domain
Helical domain
Ubiquitination site, K523
S/TQ cluster
Sample
Breast cancer
Prostate cancer
Head and neck carcinoma
Acute myeloid leukemia
Sporadic sarcoma
Colorectal cancer
Malignant pleural mesothelioma
Variant effect
Missense
Nonsense
Frameshift
Splicing

Fig. S7. Schemata of *FANCI* gene showing rare variants (VAF<1%) reported in the literature in (a) BC (b) and other cancers. FANCI domains were adapted from pfam (https://pfam.xfam.org). *FANCI* exon locations adapted from University of California Santa Cruz Genome Browser (https://genome.ucsc.edu).
