# Supplementary material for: A functionally impaired missense variant identified in French Canadian families implicates FANCI as a candidate ovarian cancer-predisposing gene
Source: Genome Med. 2021 Dec 3;13:186. doi: 10.1186/s13073-021-00998-5 (PMC8642877; doi:10.1186/s13073-021-00998-5)

Full blots associated with Figure 2.a

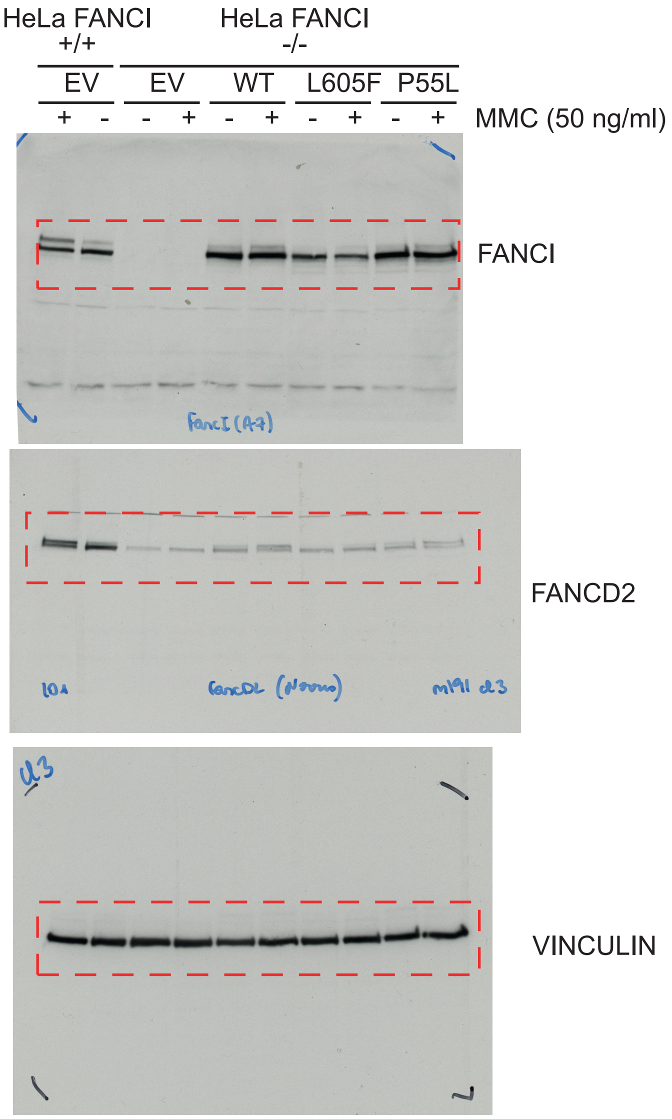

Full blots associated with Figure 2.b

Input

EV WT L605F

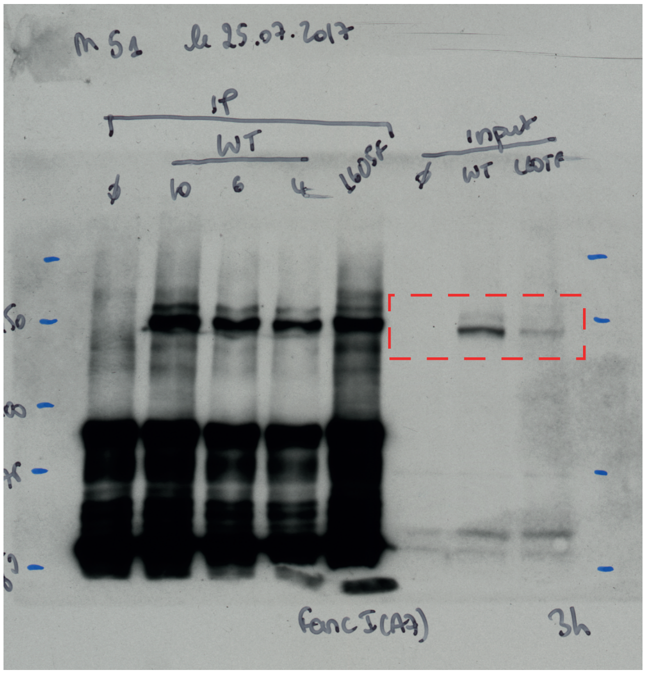

FANCI

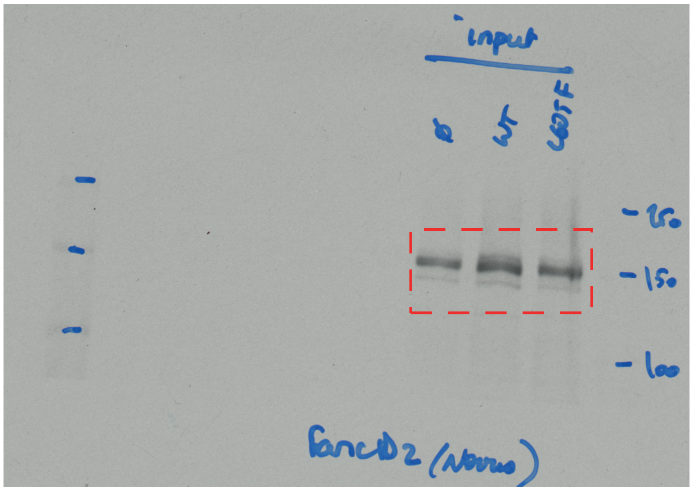

FANCD2

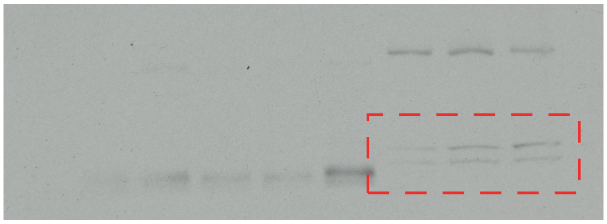

Aspecific signal

IP

EV WT L605F

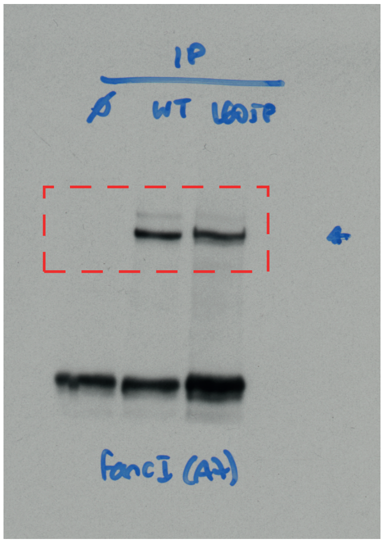

FANCI

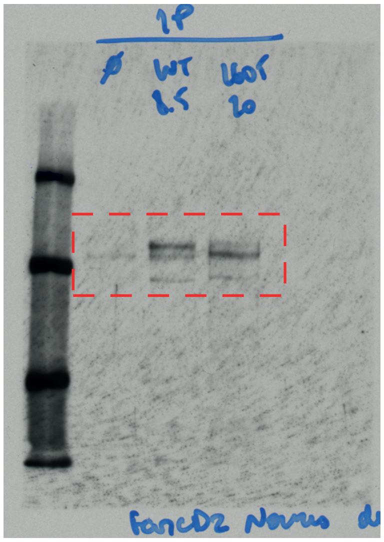

FANCD2

Full blots associated with Figure 2.d

CHX + No treatment

HeLa FANCI$^{-/-}$ + WT　　HeLa FANCI$^{-/-}$ + L605F

0　1.5　3　4　5　6　8　0　1.5　3　4　5　6　8

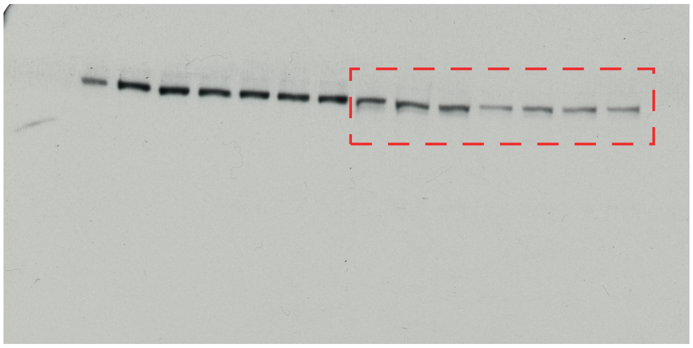

FANCI for L605F final figure

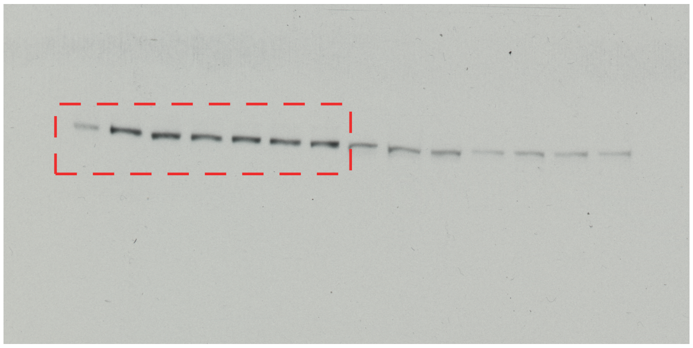

FANCI for WT final figure

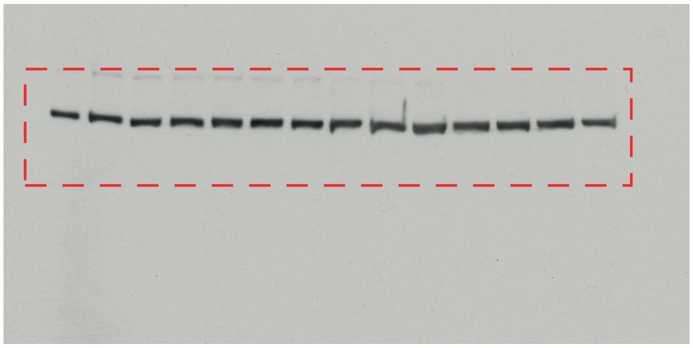

VINCULIN for both WT and L605F final figure

HeLa FANCI$^{-/-}$ + P55L

0　1.5　3　4　5　6　8

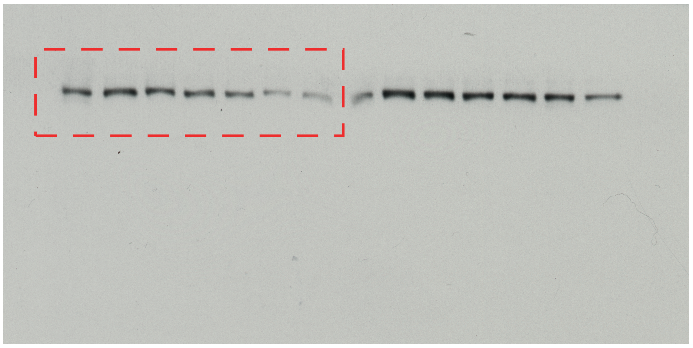

FANCI for P55L final figure

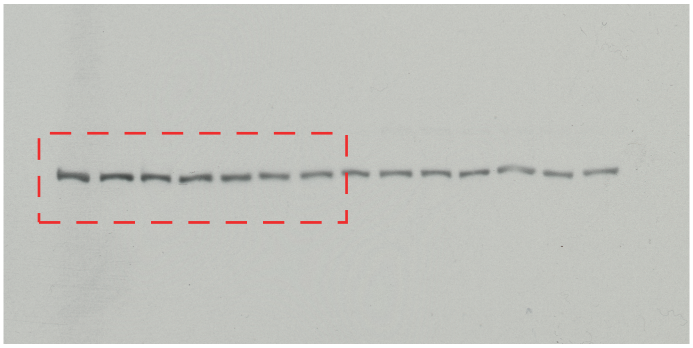

VINCULIN for P55L final figure

HeLa FANCI$^{+/+}$ + EV

0　1.5　3　4　5　6　8

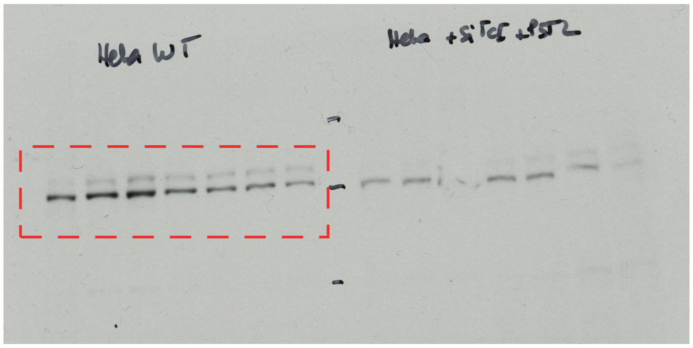

FANCI for final figure

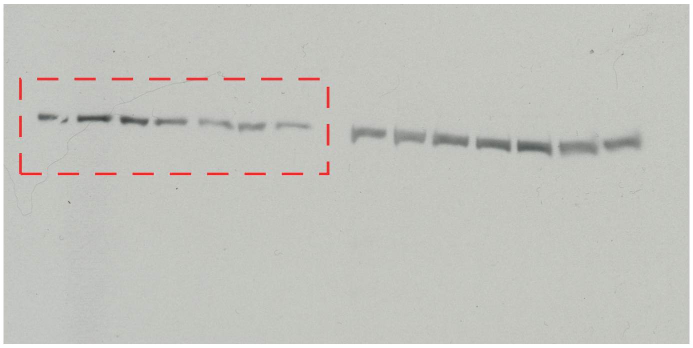

VINCULIN for final figure

Full blots associated with Figure 2.e

CHX + Formaldehyde 300 μM

HeLa FANCI$^{-/-}$ + WT — HeLa FANCI$^{-/-}$ + L605F

0 1.5 3 4 5 6 8 0 1.5 3 4 5 6 8

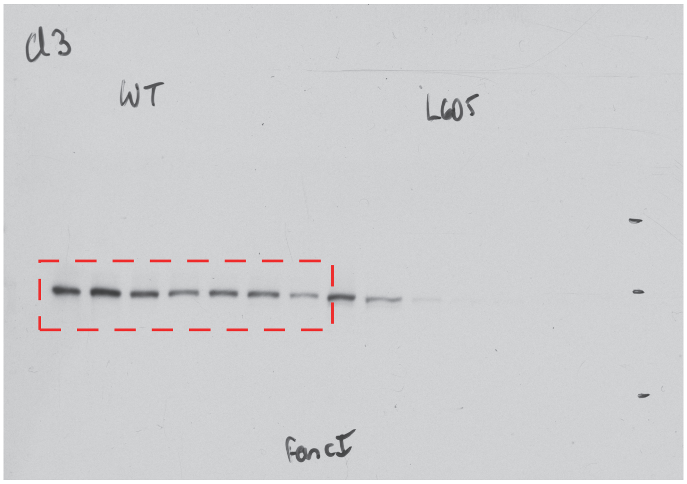

FANCI for WT final figure

HeLa FANCI$^{+/+}$+ EV — HeLa FANCI$^{-/-}$ + P55L

0 1.5 3 4 5 6 8 0 1.5 3 4 5 6 8

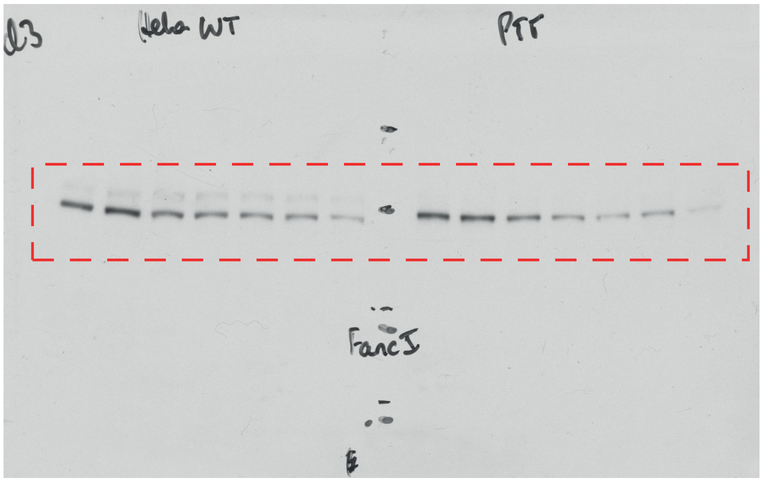

FANCI for both HeLa Fanci+/+ and P55L final figure

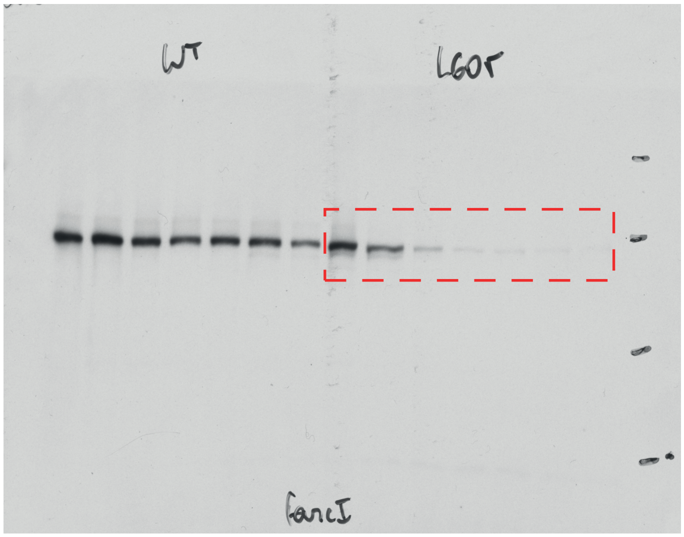

FANCI for L605F final figure

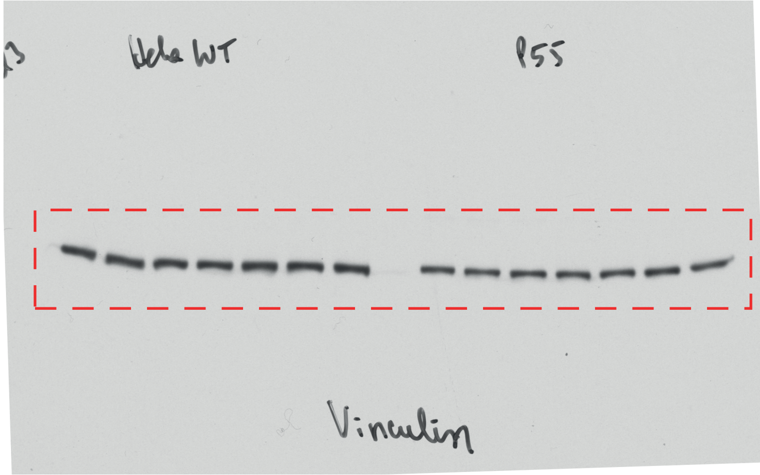

VINCULIN for both HeLa Fanci+/+ and P55L final figure

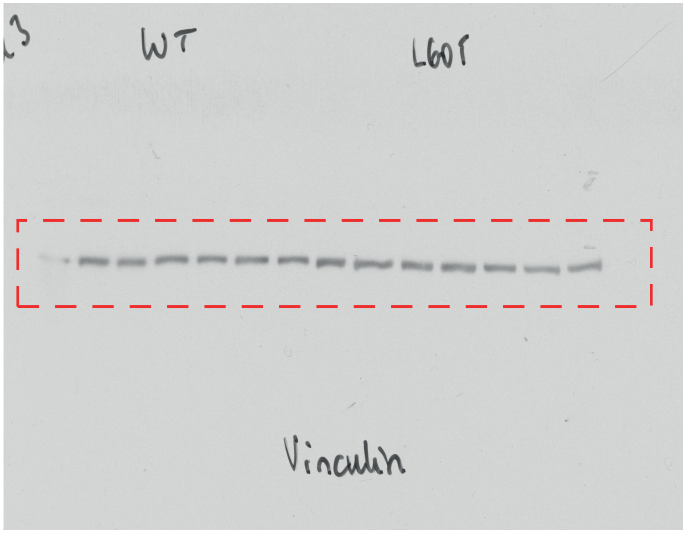

VINCULIN for both WT and L605F final figure

Full blots associated with Figure 2.f

CHX + Mitomycin C 50 ng/ml

HeLa FANCI$^{+/+}$ + EV

0 1.5 3 4 5 6 8

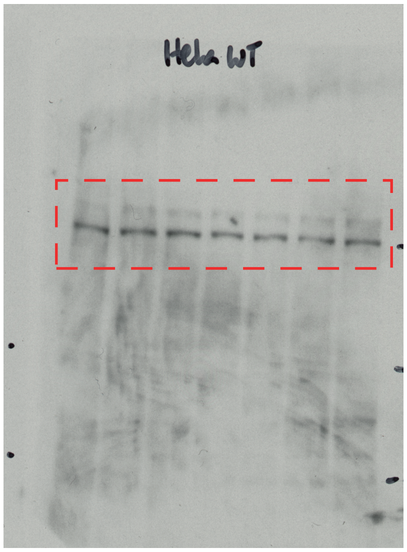

FANCI for final figure

HeLa FANCI$^{-/-}$ + WT HeLa FANCI$^{-/-}$ + L605F

0 1.5 3 4 5 6 8 0 1.5 3 4 5 6 8

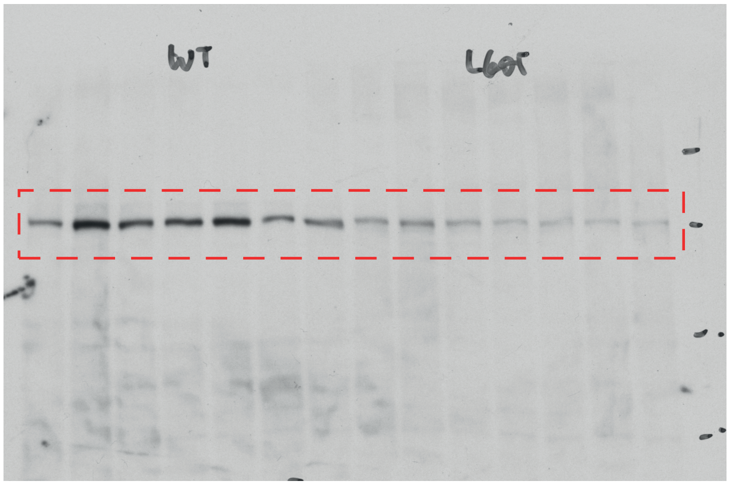

FANCI for both WT and L605F final figure

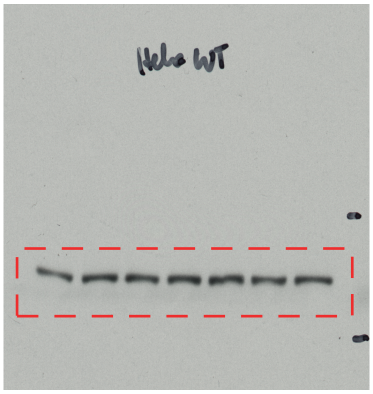

VINCULIN for final figure

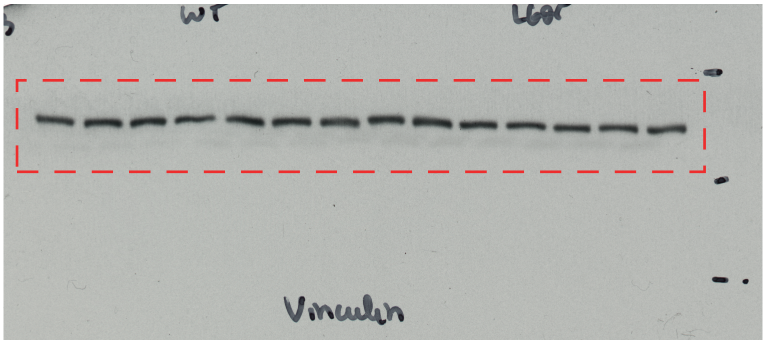

VINCULIN for both WT and L605F final figure

HeLa FANCI$^{-/-}$ + P55L

0 1.5 3 4 5 6 8

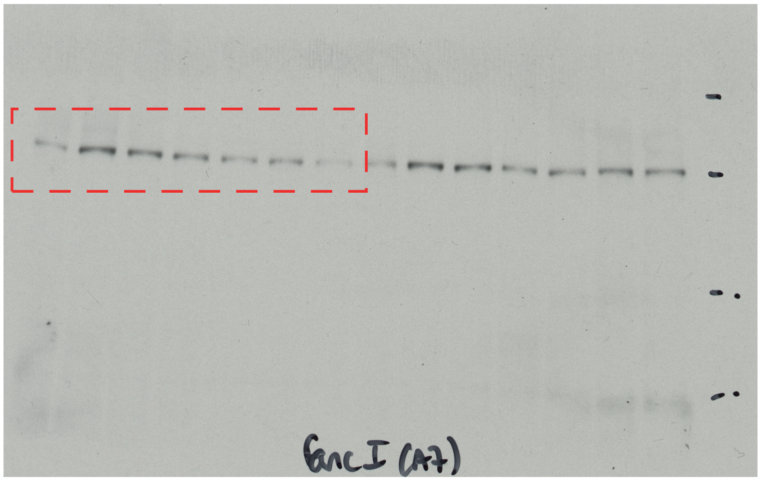

FANCI for P55L final figure

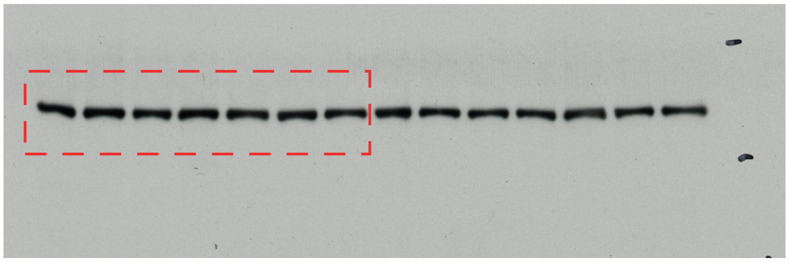

VINCULIN for P55L final figure

Full blots associated with Figure 2.g

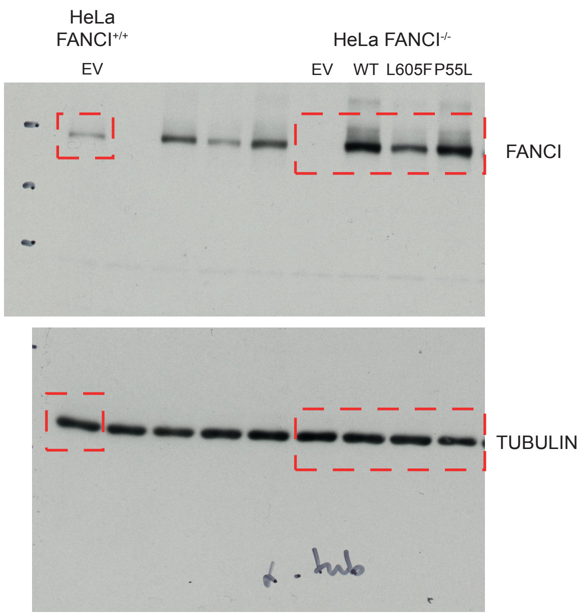

Full blots associated with Fig S1.a

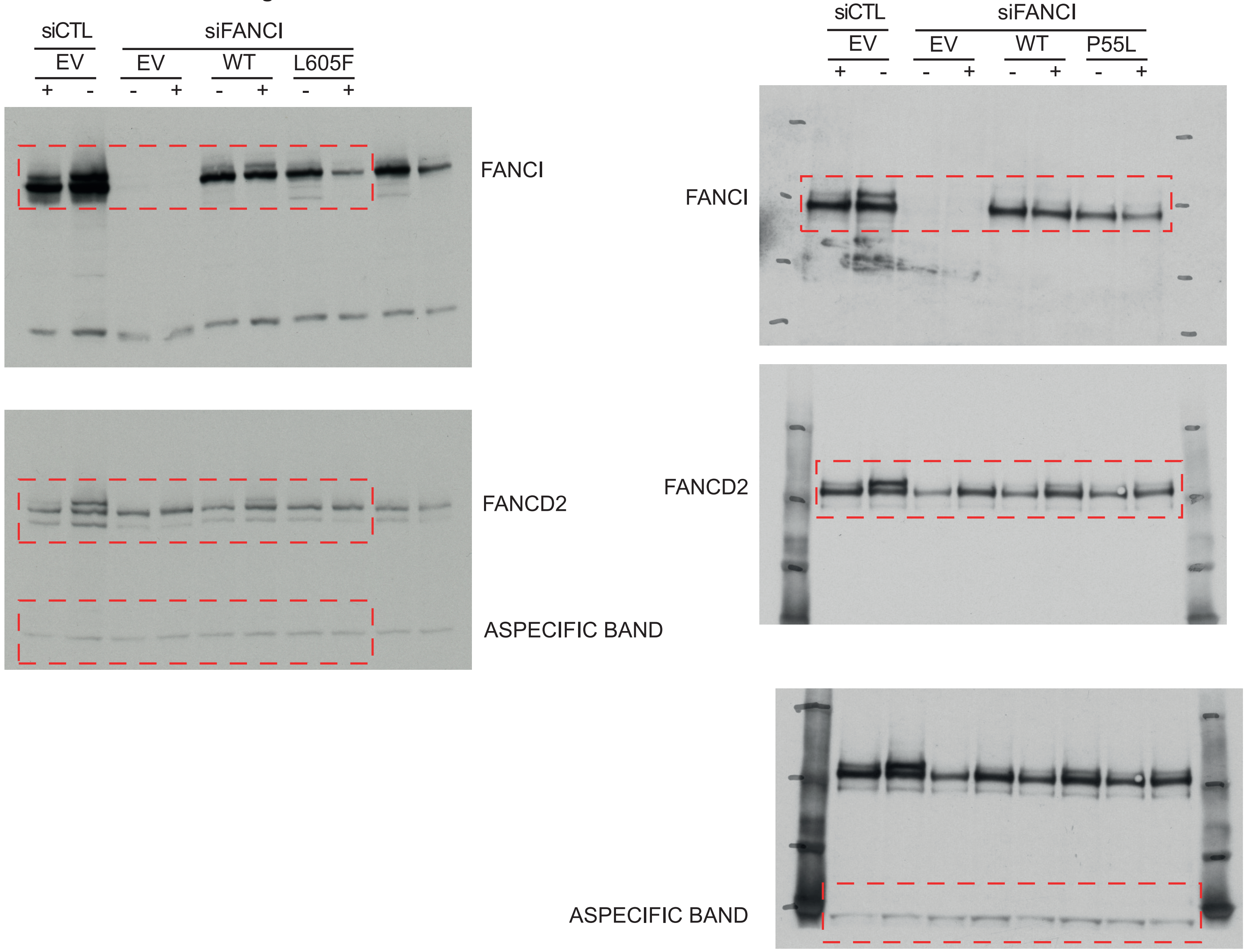

Full blots associated with Fig S1.b

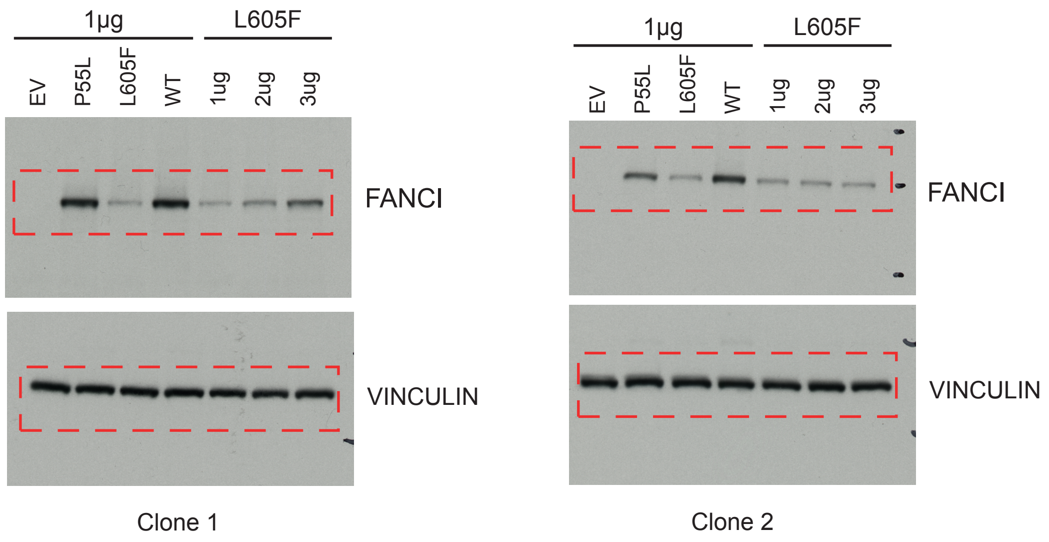

Full blots associated with Fig S1.d

CHX + No treatment

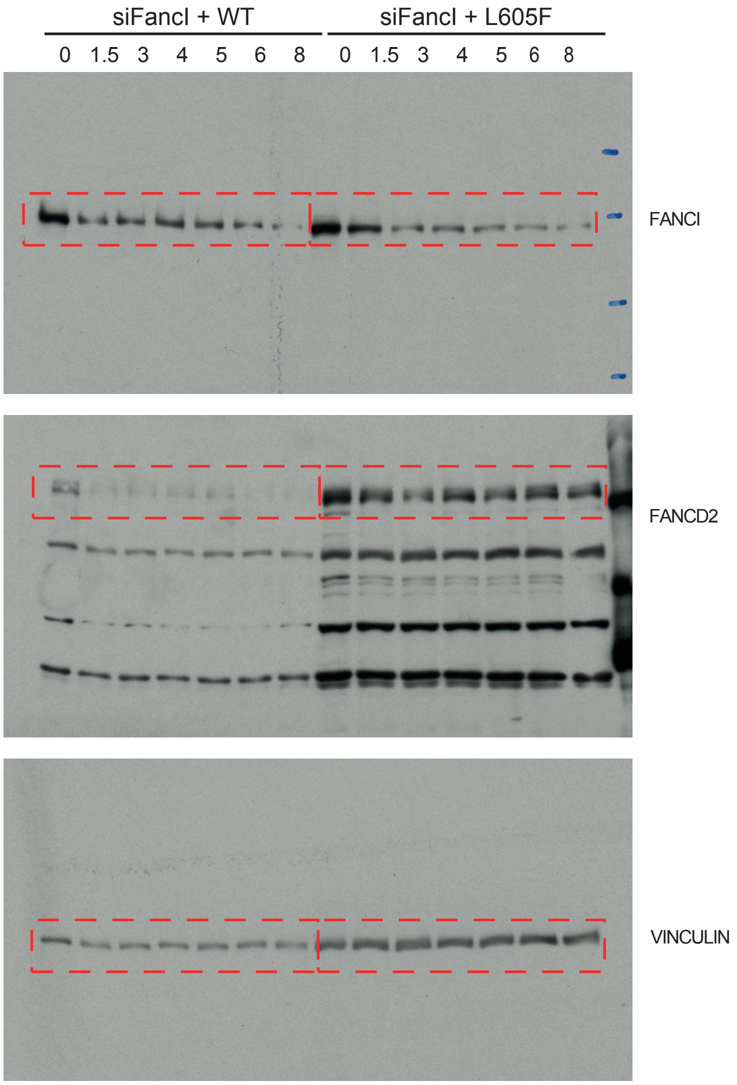

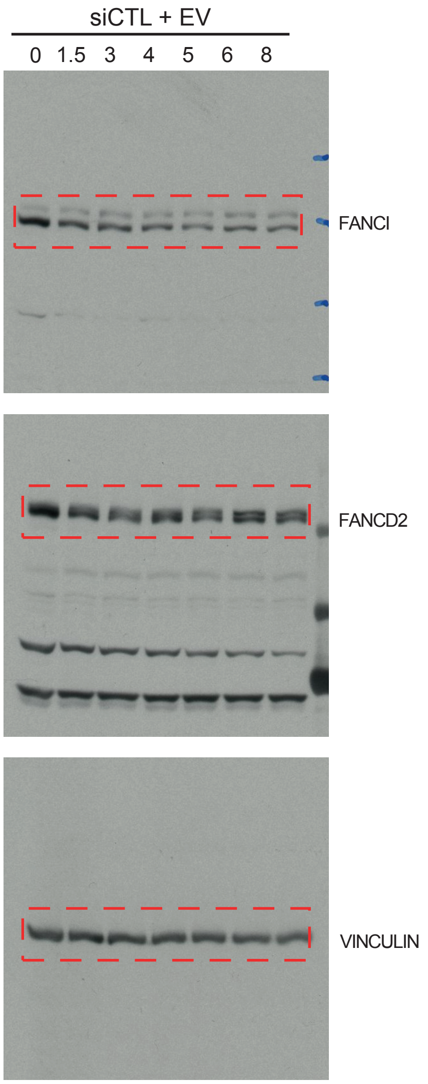

Full blots associated with Fig S1.d

CHX + Formaldehyde 300 μM

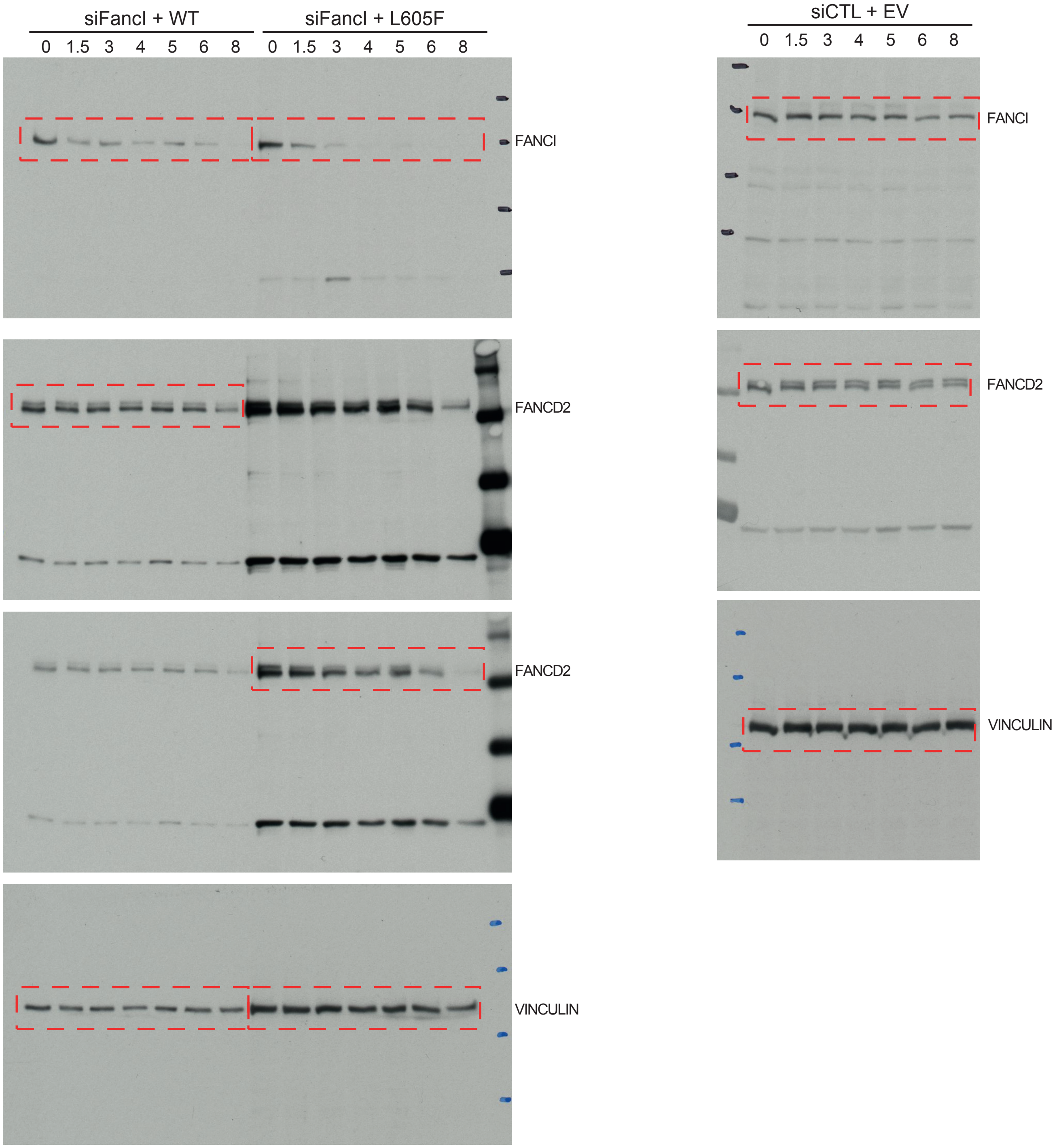

Full blots associated with Fig S1.d CHX + Mitomycin C 50 ng/ml

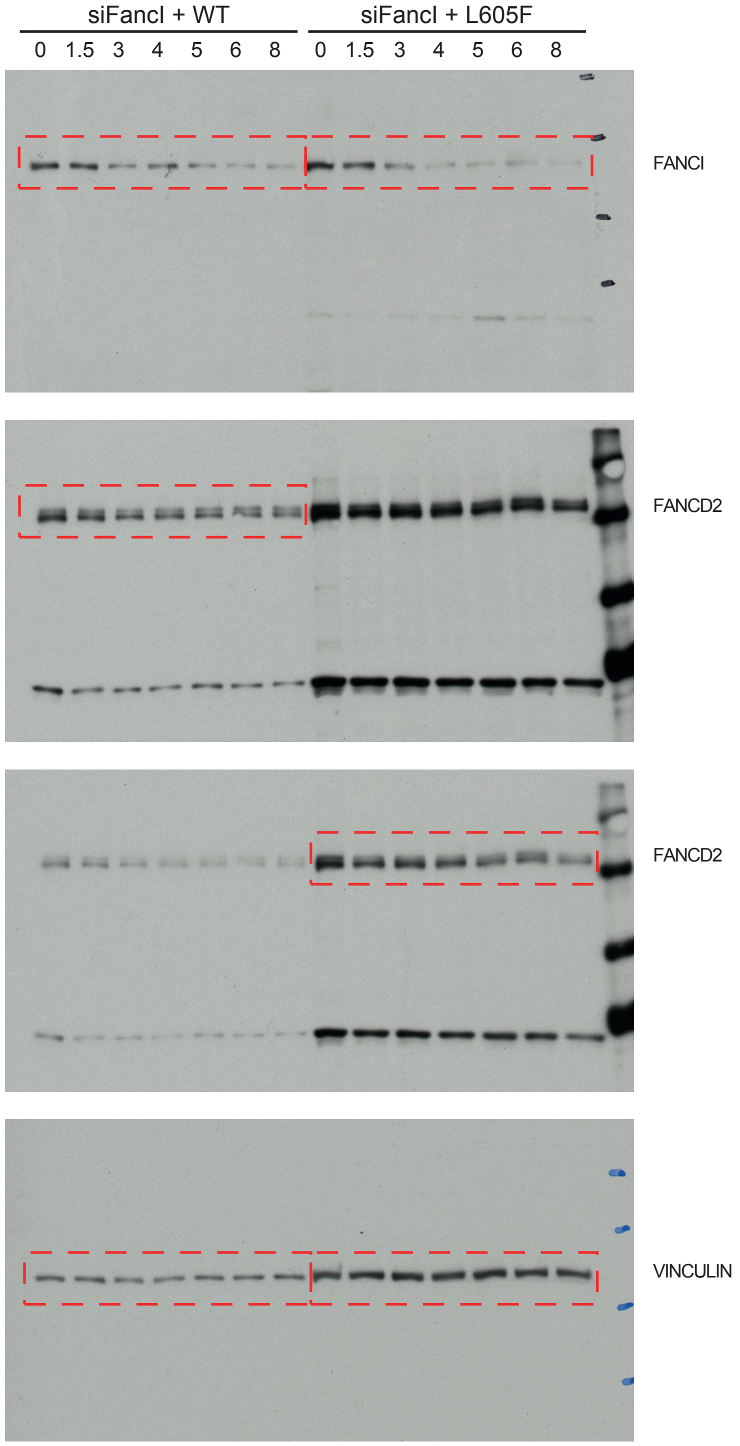

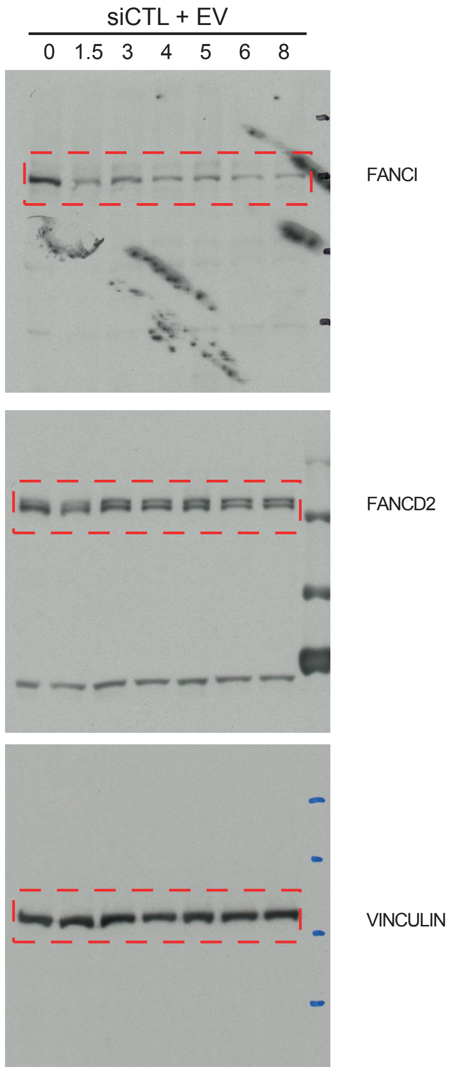

Full blots associated with Fig S2.a

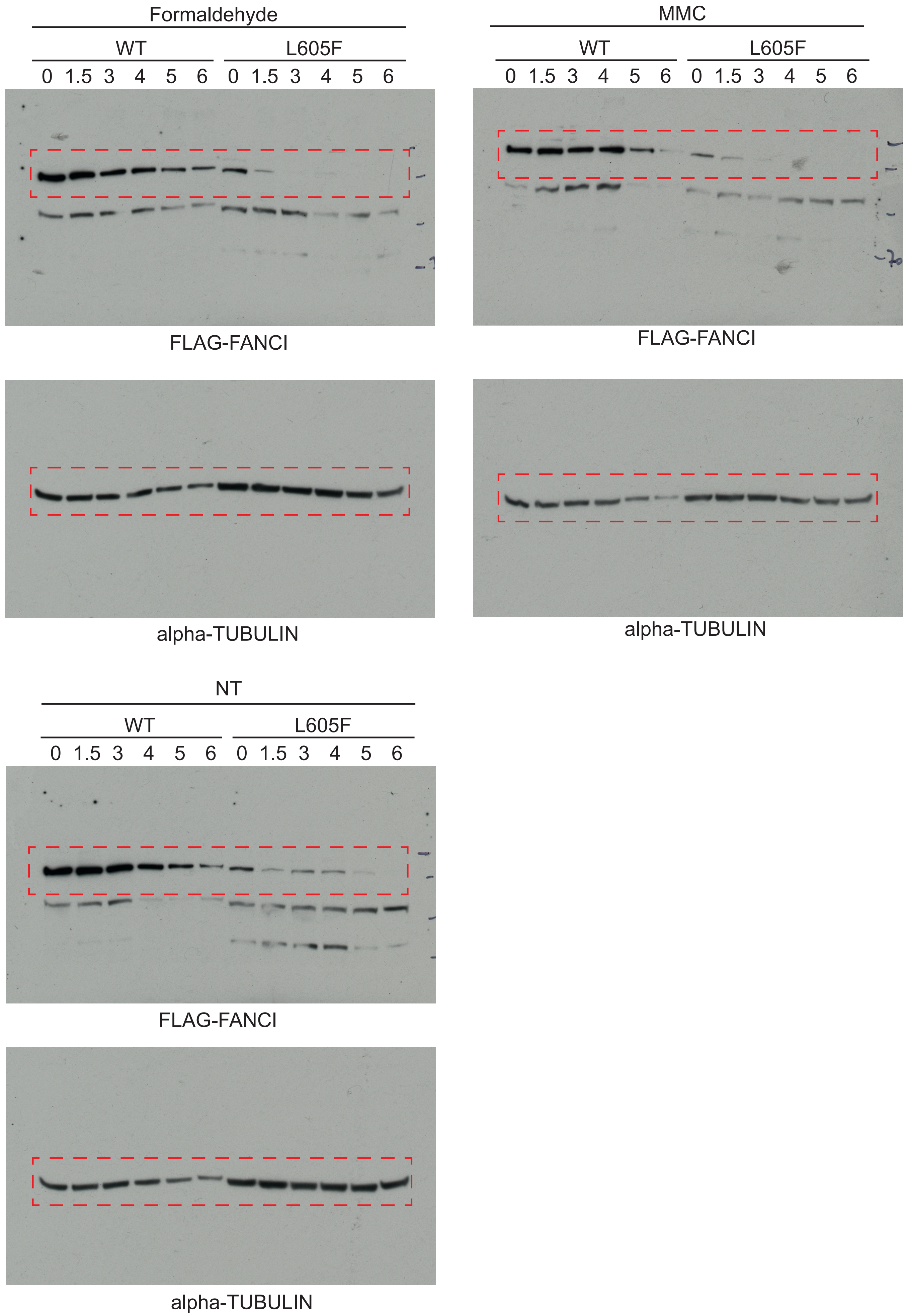

Full blots associated with Fig S2.b

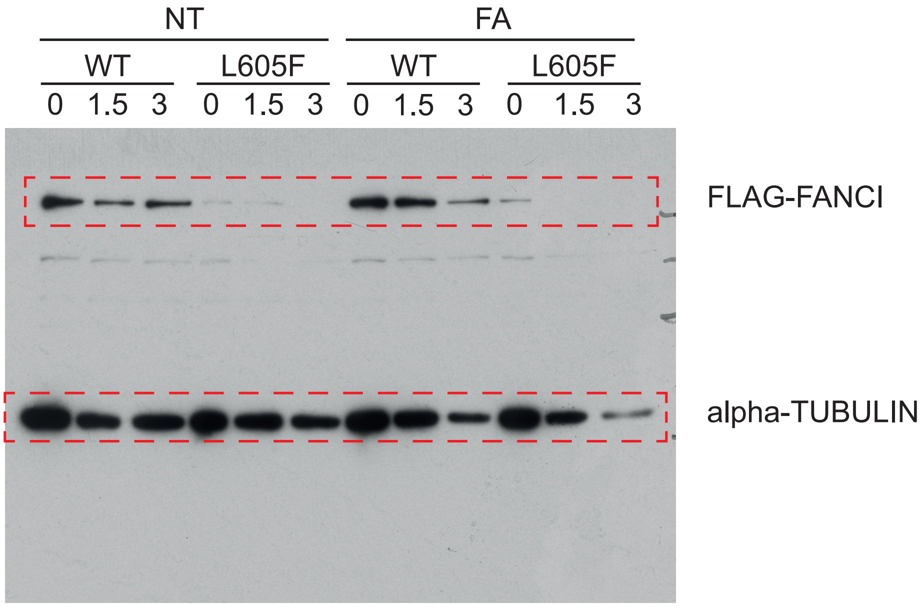

Supplement: Supplementary file 4 — Additional file 4. All full blots associated with Figure 2, Fig, S1, and Fig. S2. [file 13073_2021_998_MOESM4_ESM.pdf]
